# Upper-tropospheric bridging of wintertime surface climate variability in the Euro-Atlantic region and northern Asia

**DOI:** 10.1038/s41598-019-51019-w

**Published:** 2019-10-10

**Authors:** Pawel Schlichtholz

**Affiliations:** Institute of Oceanology, Polish Academy of Sciences, Powstancow Warszawy 55, 81-712 Sopot, Poland

**Keywords:** Atmospheric dynamics, Climate and Earth system modelling

## Abstract

A remarkable feature of interannual climate variability is a robust link of wintertime anomalies of surface air temperature (SAT) in northern Asia to pan-Atlantic SAT variations associated with the North Atlantic Oscillation (NAO). Here statistical analyses of data from the era of satellite observations (1979–2017) are used to show that about 80% of the variance of the winter (December-March) mean area-averaged SAT anomalies in northern Asia can be explained by the anomalous surface circulation associated with an NAO-like mode of sea level pressure variability over extratropical Eurasia. These SAT anomalies are related equally strongly to the “Lake Baikal” vortex representing variations of the upper-tropospheric circulation over northern Asia. Support is given for the scenario that this vortex drives SAT anomalies in northern Asia via surface-reaching displacements of isentropic surfaces and that it is coupled to climate variability in the Euro-Atlantic sector via interactions between the North Atlantic storm track, quasi-stationary planetary waves, and zonal-mean zonal winds. The results underpin the importance of a lesser-known zonal wavenumber-3 structure of disturbances trapped over Eurasia by the polar front jet rather than the better-known zonal wavenumber-5 structure of disturbances trapped by the subtropical jet for NAO teleconnections.

## Introduction

**Figure 1:**
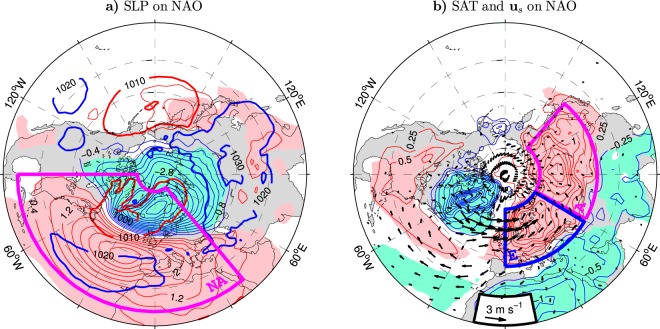
Relation of wintertime (DJFM mean) surface climate variability in the NH extratropics to the NAO in the ESO period. (**a**) Anomalies of the sea level pressure (thin contours and color shading) regressed onto the NAO index defined as the PC time series of the leading mode of SLP variability in the North Atlantic region (magenta box). (**b**) Anomalies of the surface air temperature (thin contours and color shading) and surface wind **u**_*s*_ (arrows) regressed onto the NAO index. In (**a** and **b**) red (blue) contours represent positive (negative) anomalies. The contour interval (CI) is 0.4 hPa and 0.25 K per unit NAO index, respectively. The zero contour is omitted. Pink (aquamarine) shading denotes positive (negative) anomalies statistically significant at the 95% confidence level. In (**a**) thick contours show the wintertime climatology of SLP (in hPa). In (**b**) the anomalies of **u**_*s*_ (subsampled and masked if both components are nonsignificant at the 95% confidence level) are in m s^−1^ per unit NAO index. The blue and magenta boxes delineate areas for which indices of SAT variability are constructed. The maps were generated by MathWorks MATLAB R2014a with M_Map (http://www.eoas.ubc.ca/~rich/map.html).

Climatic anomalies are usually defined as departures of primary climate variables, such as the sea level pressure (SLP) or surface air temperature (SAT), averaged over a specific (monthly, seasonal, annual or any other) timespan from their climatological means or secular trends. At a given time, these anomalies are characterised by spatial patterns exhibiting distinct lobes (often of opposite sign) that typically cover large geographic regions in distant parts of the globe. These patterns of simultaneous climate variations, referred to as oscillations, modes or teleconnections^1,2^, are recurrent but possess no particular periodicity. In the Northern Hemisphere (NH) extratropics, especially during the coldest part of the year, the most prominent teleconnection pattern is the North Atlantic Oscillation (NAO). The NAO is related to quasi-meridional displacements of atmospheric mass between the high and the middle latitudes of the Atlantic sector. During the positive polarity of the NAO, a lobe of below-normal SLPs in the Arctic is accompanied by a lobe of above-normal SLPs south of about 55°N^3,4^. This anomalous dipole is illustrated by the thin contours and color shading in Fig. 1a showing the NAO-covariant pattern of the wintertime (DJFM mean) SLP anomalies in the NH extratropics for the era of satellite observations (ESO period; here winters 1980–2017, years of the January) based on data from the National Centers for Environmental Prediction/National Center for Atmospheric Research (NCEP/NCAR) reanalysis^5^. The NAO dipole of SLP anomalies corresponds to enhanced meridional pressure gradients between its lobes (see the thick contours in Fig. 1a for the wintertime SLP climatology in the ESO period). As a result, it drives stronger than normal westerly winds over the subpolar North Atlantic and northern Europe (see the arrows in Fig. 1b for the significant NAO-covariant wintertime surface wind anomalies in the ESO period). Conversely, in the negative phase of the NAO, the SLPs in the Arctic are enhanced while the SLPs in the south are reduced so that the surface westerlies onto Europe are weaker than normal.

The largest anomalies in the northern and southern lobes of the NAO-covariant SLP dipole appear slightly northeast of the climatological “centers of action” of the Icelandic Low and the Azores High, respectively (Fig. 1a). Therefore, commonly used simple indices of the NAO are based on the SLP difference between the Azores or Lisbon and Iceland^3,6,7^. These station-based indices are generally consistent with alternative NAO indices extracted from gridded SLP or geopotential height (GPH) data using the empirical orthogonal function (EOF) technique. The EOF technique enables one to decompose the anomaly field into a set of mathematically independent (orthogonal) spatial patterns, each associated with a time series usually referred to as the principal component (PC) time series (see Methods). This technique singles out the NAO mode as the leading EOF (the one which explains the largest fraction of the variance in the data) of SLP variability computed over a more or less restricted Atlantic domain^4^. In the ESO period, for instance, the PC-based wintertime NAO index derived from SLP data in the North Atlantic region (NA box in Fig. 1a) correlates with the standard station-based NAO index^3^ at the level of $r=0.96$^8^. The anomaly patterns in Fig. 1 are obtained by regressions just upon the PC-based NAO index (shown in Supplementary Fig. S1a, red curve).

The NAO varies on a broad range of timescales from submonthly^9^ to multidecadal^3,10^. On all timescales, it is intimately related to the location and intensity of the North Atlantic jet stream (a high-speed core of predominantly westerly mean winds in the upper troposphere) and the North Atlantic storm track (weather systems moving towards Europe)^11–16^. This relationship has, however, different characteristics on different timescales. On the multidecadal scale, for instance, the NAO variability is mainly associated with changes in the strength of the jet and storm track. On the interannual-to-decadal scale, it is dominated by their spatial displacements^10^. In the ESO period, the interannual variability of the wintertime NAO occurs in tune with meridional displacements and zonal excursions of the North Atlantic storm track^8^.

Swings of the NAO from one phase to another and the related storm track variations bring about large changes in SAT and precipitation over the North Atlantic and across vast areas of the adjacent continents^4,12,17^. Historically (see ref.^18^ for a review), the best-known NAO climate impact is a seesaw in winter temperatures between northern Europe and western Greenland (see the thin contours and color shading in Fig. 1b for the NAO-covariant wintertime SAT anomaly pattern in the ESO period). As the seesaw is quite robust, the east-west contrast between SAT across the subpolar North Atlantic was even considered as an index of the NAO variability^19,20^. In addition to the Europe-Greenland temperature seesaw, the NAO-related SAT anomaly patterns exhibit significant out-of-phase temperature variations over North Africa and the Middle East (cooling when northern Europe is warmed) and southeastern North America (warming when the Greenland-Labrador region is cooled). A remarkable feature of these patterns is the presence of significant temperature anomalies over northern Asia that are in phase with the temperature anomalies over northern Europe. This feature is characteristic for the NAO-covariant temperatures in the ESO period (Fig. 1b) as well as longer periods^21–23^. It also appears in the anomaly patterns of lower-tropospheric temperatures associated with a hemispheric mode of variability that is closely related to the NAO and known as the Arctic Oscillation (AO)^24–26^.

Mechanisms of the NAO/AO influence on Asian temperatures are complex and not fully understood. They may involve temperature advection by anomalous zonal-mean winds^25^ as well as interactions of the NAO with the Ural blocking^27^, the Siberian High, and the East Asian monsoon (see refs^28,29^ for reviews). Low-frequency teleconnections are often established via the waveguiding effect of the time-averaged upper-tropospheric jets^30^. This effect produces zonally oriented chains of perturbations governed by planetary (Rossby) waves dynamics. In winter, these perturbations have a circumglobal character^31,32^ and contribute to the NAO^33,34^ and AO^35,36^ variability. Quasi-stationary planetary waves play a key role, for instance, in bridging the winter East Asian monsoon to the NAO/AO and the associated anomalies of the zonal-mean circulation in the upper troposphere/lower stratosphere^29,37,38^.

The studies on the NAO relation to the circumglobal wavetrains underscore the importance of patterns of disturbances with a zonal wavenumber-5 structure that is related to the waveguiding effect of the subtropical Asian jet^32–34,39^. However, this effect may not provide an adequate explanatory framework for the relation between the NAO and SAT anomalies in northern Asia. On subseasonal timescales, these anomalies are occasionally related to an anomalous upper-tropospheric circulation in the Lake Baikal area, which is linked to the NAO via planetary waves propagating through Europe from the North Atlantic region^40^. On the seasonal timescale, the wintertime circulation and air temperature anomalies in the Lake Baikal area are often related to the NAO via changes in the strength of the stratospheric polar night jet^41^. A quite robust relation of the NAO to the anomalous upper-tropospheric circulation in the Lake Baikal area, hereafter referred to as the “Lake Baikal” vortex, was reported in a recent study of the winter mean circulation in the ESO period^8^. Specifically, that study showed a strong association between the “Lake Baikal” vortex and the leading EOF mode of the wintertime storm track activity (STA) over Eurasia, hereafter referred to as the STA_EA_ mode, that is closely related to the NAO. It also showed that the STA_EA_-related “Lake Baikal” vortex is embedded in a wavenumber-3 circumglobal waveguide pattern (CWP3) guided by the polar front jet. These results suggest that interactions between the North Atlantic storm track and high-latitude stationary waves are instrumental in the linkage of SAT anomalies in northern Asia to the NAO.

While high-latitude CWP3s may significantly contribute to subseasonal and multidecadal surface climate variability^31^, their role in bridging climate anomalies in Asia to the storm track/NAO variability in the Euro-Atlantic sector has not been explored yet. The present study expands upon the findings on the wintertime climate variability in Eurasia reported in the already mentioned related study^8^. The related study mainly focused on the predictability of the storm track/NAO variability from Arctic sea ice cover anomalies. The present study examines in greater detail the relation of air temperature anomalies in northern Asia to the concurrent variability of the large-scale atmospheric circulation and storm tracks, as well as the role of quasi-stationary planetary waves and zonal-mean zonal wind anomalies in maintaining this relation. Unless stated otherwise, the results are based on statistical analyses of linearly detrended data from the NCEP/NCAR reanalysis in the ESO period (see Supplementary Table S1 for a summary of acronyms used in the study).

## Relation of Air Temperature Anomalies in Northern Asia to Variations in Tropospheric Circulation

### Relation to surface circulation anomalies

Correlation coefficients between key indices of the wintertime climate variability over extratropical Eurasia during the ESO period are given in Table 1. Indices of air temperature variations are based on SAT anomalies at the latitudes (40°–70°N) of the broad northern lobe of significant NAO-covariant SAT anomalies extending across the Eurasian continent between its Atlantic and Pacific coasts (Fig. 1b). Two distinct cores appear in this lobe, one in northern Europe and one in northern Asia. Air temperature variations over Europe and northern Asia are represented by the SAT_E_ and SAT_A_ indices defined as standardised anomalies of SAT averaged over the blue and magenta boxes in Fig. 1b, respectively. The analogous measure of air temperature variations over entire northern Eurasia (the SAT_E+A_ index) is based on SAT anomalies averaged over both boxes. Even though only the western part of Eurasia is under the direct influence of strong NAO-covariant surface wind anomalies (Fig. 1b), the NAO relation to air temperatures in northern Asia is as pronounced as its impact on air temperatures in Europe. Indeed, the correlations of the NAO index with the SAT_A_ and SAT_E_ indices are equally high ($r=0.78$).

**Table 1:** Wintertime correlation coefficients (×100) of the indices of surface air temperature variability over Europe (SAT_E_), northern Asia (SAT_A_) and northern Eurasia (SAT_E+A_) with the NAO index (PC1 of sea level pressure variations in the North Atlantic region, NA box in Fig. 1a), SLP_EA_ index (PC1 of sea level pressure variations over extratropical Eurasia, EA box in Fig. 2b), GPH_LB_ index (area-averaged geopotential height anomalies at 300 hPa over the Lake Baikal area, LB box in Fig. 2c) and STA_EA_ index (PC1 of upper-tropospheric storm track activity (${\overline{v^{\prime} v^{\prime} }}_{300}$) variations over extratropical Eurasia, EA box in Fig. 6b).

Index	NAO	SLP_EA_	GPH_LB_	STA_EA_
SAT_E_	***78***	***75***	***60***	***72***
SAT_A_	***78***	***91***	***89***	***80***
SAT_E+A_	***82***	***89***	***81***	***81***

The SAT_E_, SAT_A_, and SAT_E+A_ indices represent the area-averaged temperature within the E, A, and E+A boxes in Fig. 1b, respectively. The PC1s are the first principal component time series from the EOF decomposition of the given variable in the given area. All indices are based on linearly detrended DJFM mean data in the ESO period (1980–2017, years of the January). All correlations are significant at the 99.9% confidence level.

**Figure 2:**
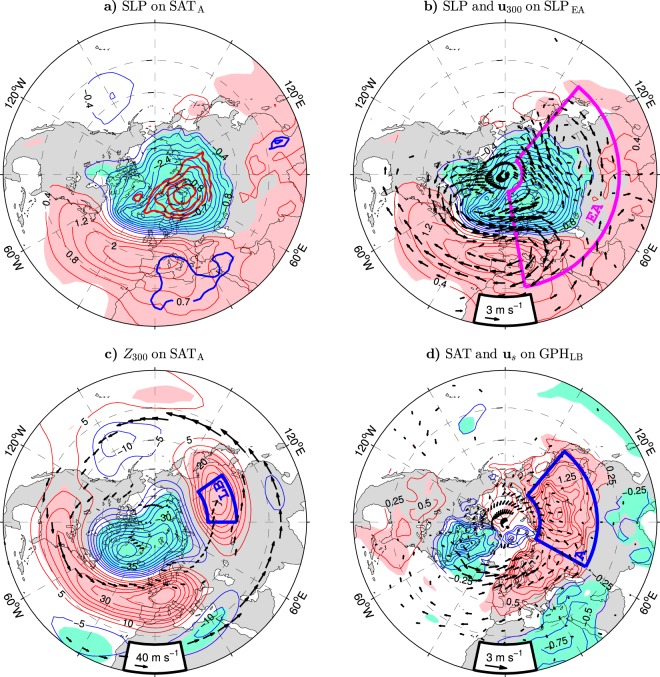
Wintertime surface and upper-tropospheric anomalies in the NH extratropics associated with indices of Eurasian climate variability in the ESO period. (**a** and **b**) Anomalies of the sea level pressure (thin contours and color shading) regressed onto the SAT_A_ and SLP_EA_ indices, respectively. The CI is 0.4 hPa per unit index. In (**a**) thick contours are the isolines of high correlations ($|r|=\{0.70,0.75,0.80,0.85\}$). In (**b**) arrows show the SLP_EA_-covariant anomalies of the horizontal wind velocity (in m s^−1^ per unit SLP_EA_ index, subsampled and masked if both components are nonsignificant at the 95% confidence level) at 300 hPa. (**c**) Anomalies of the geopotential height at 300 hPa (*Z*_300_, thin contours and color shading) regressed onto the SAT_A_ index. The CI is 5 gpm per unit SAT_A_ index. Black arrows depict the wintertime climatology of jet streams. (**d**) Anomalies of the surface air temperature (thin contours and color shading) and surface wind **u**_*s*_ (arrows) regressed onto the GPH_LB_ index. The CI is 0.25 K per unit GPH_LB_ index. The anomalies of **u**_*s*_ (subsampled and masked if both components are nonsignificant at the 95% confidence level) are in m s^−1^ per unit GPH_LB_ index. The contour and shading colors used in (**a**–**d**) are explained in the caption to Fig. 1. The SAT_A_, SLP_EA_ and GPH_LB_ indices are defined in the caption to Table 1. The maps were generated by MathWorks MATLAB R2014a with M_Map (http://www.eoas.ubc.ca/~rich/map.html).

Given the close relation of air temperatures in northern Asia to the NAO, the pattern of SLP anomalies regressed onto the SAT_A_ index (thin contours and color shading in Fig. 2a) is similar to the NAO-covariant pattern of these anomalies (Fig. 1a). Higher-than-normal air temperatures in northern Asia are associated with reduced SLPs in the Arctic and increased SLPs in the middle latitudes of the Euro-Atlantic sector. However, compared to the NAO-covariant SLP dipole, the centers of action in the SAT_A_-covariant SLP dipole are shifted eastward. The northern (stronger) center appears over the Barents Sea, and the southern (weaker) center extends to southwestern Europe. Highly significant anomalies are found on the eastern side of the northern center. In the southeastern Barents Sea/southern Kara Sea region, the correlations of the SLP anomalies with the SAT_A_ index exceed 0.85 (see the thick contours in Fig. 2a). These high correlations underscore the importance of processes that tend to extend the Icelandic Low along the Iceland-Barents Sea corridor for climate variability in northern Asia.

The pattern of the SAT_A_-covariant SLP anomalies in Fig. 2a bears a striking resemblance to the pattern of SLP anomalies regressed onto the PC time series of the leading mode of SLP variability over extratropical Eurasia (EA box in Fig. 2b), hereafter referred to as the SLP_EA_ index (see the thin contours and color shading in Fig. 2b for the SLP_EA_-covariant SLP anomaly pattern). The SLP_EA_ index correlates with the NAO index at 0.83 and the SAT_A_ index at 0.91 (see Supplementary Fig. S1a,b for comparison of the time series). These correlations are consistent with a slightly stronger dependence of temperature variations in northern Eurasia (the SAT_E+A_ index) on the SLP_EA_ index ($r=0.89$) than the NAO index ($r=0.82$).

**Figure 3:**
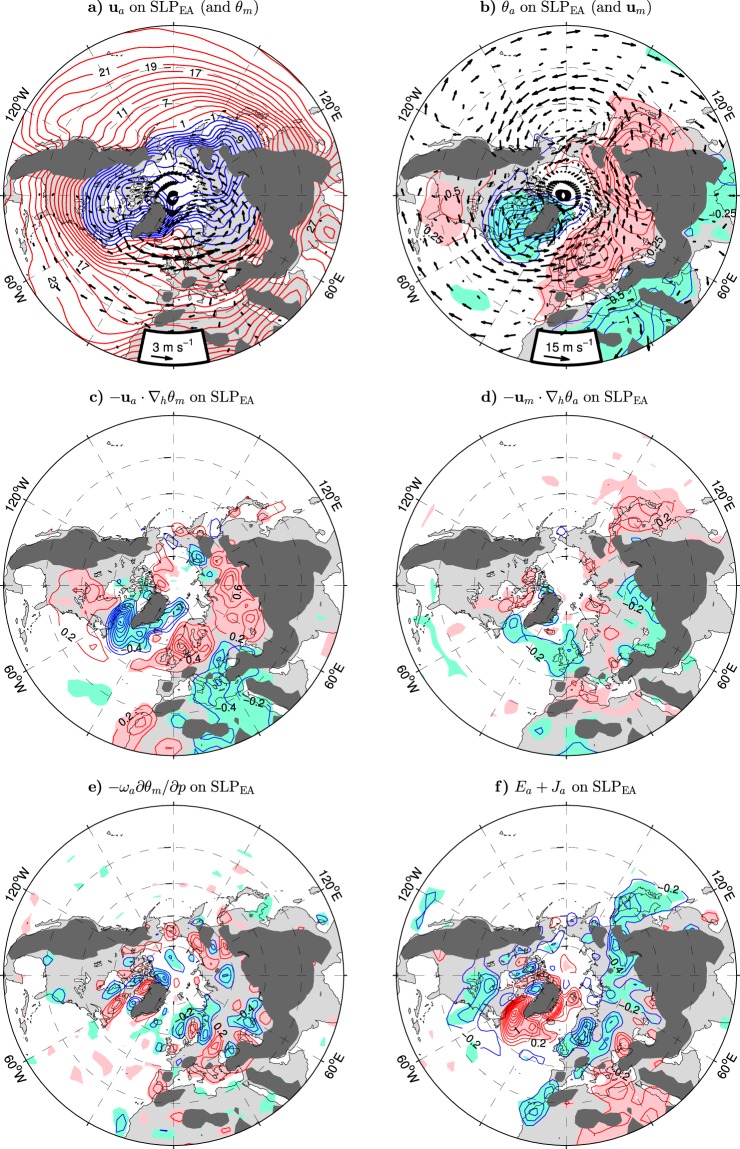
Wintertime atmospheric anomalies at 925 hPa in the NH extratropics regressed onto the SLP_EA_ index defined as the PC time series of the leading mode of sea level pressure variability over extratropical Eurasia (EA box in Fig. 2b) in the ESO period. (**a**) Anomalies of the horizontal wind velocity (arrows, in m s^−1^ per unit SLP_EA_ index, subsampled and masked if both components are nonsignificant at the 95% confidence level) superimposed onto the wintertime climatology of the potential temperature (thick contours; CI: 4 °C). (**b**) Anomalies of the potential temperature (thin contours and color shading; CI: 0.25 K per unit SLP_EA_ index) on the background of the wintertime climatology of the horizontal wind velocity (arrows, in m s^−1^). (**c**) Mean temperature advection by the anomalous horizontal wind. (**d**) Anomalous temperature advection by the mean horizontal wind. (**e**) Mean temperature advection by the anomalous vertical motion. (**f**) Anomalous eddy heat flux convergence and diabatic heating. In (**c**–**f**) the CI is 0.2 K day^−1^ per unit SLP_EA_ index. In (**a**–**f**) dark shading masks approximately areas where the climatological pressure at the surface is lower than 925 hPa. The thin contour and other shading colors are explained in the caption to Fig. 1. The maps were generated by MathWorks MATLAB R2014a with M_Map (http://www.eoas.ubc.ca/~rich/map.html).

To further examine the impact of anomalous circulation on lower-tropospheric temperatures in northern Eurasia, the middle and lower panels in Fig. 3 display patterns of the SLP_EA_-covariant terms of the thermodynamic equation (Eq. 2 in Methods) at 925 hPa. To facilitate interpretation of these terms, the arrows in the upper panels of Fig. 3 show the SLP_EA_-covariant wind anomalies superimposed on the climatological temperature (Fig. 3a) and the climatological wind field superimposed on the SLP_EA_-covariant temperature anomalies (Fig. 3b) at the same level. Dark shading marks the approximate location of the mountain ranges transecting the 925 hPa level to emphasise their guiding effect on the anomalous near-surface circulation over Eurasia. Mean temperature advection by the anomalous wind is evidently a key driving agent for the near-surface NAO-like air temperature variations (compare the pattern of $-{{\bf{u}}}_{a}\cdot {\nabla }_{h}{\theta }_{m}$ in Fig. 3c with the temperature anomalies in Fig. 3b). When the SLP_EA_ index is positive, the Greenland-Labrador area is abnormally cooled by the enhanced transport of polar air by northerly and northwesterly wind anomalies while Europe is abnormally warmed by the reinforced transport of maritime air by anomalous westerlies. At the same time, Siberia is warmed due to the warm air advection by southwesterly wind anomalies. The distinct core of anomalous temperature north of Lake Baikal is induced by significant wind anomalies across a zone of strong baroclinicity (large horizontal gradients of the climatological temperature) in this area (Fig. 3a). The Asian and European cores of the anomalous temperature field are separated by the meridionally-oriented Ural Mountains at about 60°E (not seen in Fig. 3 because of their relatively small height), indicating that the topography of the Urals may play a role in forming these cores. The major mountain chains of the Eurasian continent should also be important, as suggested by the anomalous winds over eastern Europe and western Asia that follow a U-shaped route along the northern rim of these chains (Fig. 3a).

In northeastern Asia, some near-surface warming during the positive polarity of the SLP_EA_ index results from anomalous descending motion across the climatological isentropic surfaces, as indicated by the sign of mean temperature advection by the anomalous pressure velocity ($-{\omega }_{a}\partial {\theta }_{m}/\partial p$) at 925 hPa in this area (Fig. 3e). Farther south, along the Pacific coast, the SLP_EA_-covariant temperature anomalies are primarily due to anomalous temperature advection by the climatological wind (see Fig. 3d for the pattern of $-{{\bf{u}}}_{m}\cdot {\nabla }_{h}{\theta }_{a}$ at 925 hPa). The combined forcing by anomalous eddy heat flux convergence (*E*_*a*_) and diabatic heating (*J*_*a*_) tends to destroy the SLP_EA_-covariant temperature anomalies in northern Europe as well as in northern Asia, as indicated by the sign of ${E}_{a}+{J}_{a}$ at 925 hPa in these areas (Fig. 3f).

### Relation to upper-tropospheric circulation anomalies

Above-normal air temperatures over northern Asia are associated with a poleward shift of the North Atlantic jet stream. This shift is illustrated in Fig. 2c showing the anomaly pattern of GPH at 300 hPa (*Z*_300_) regressed onto the SAT_A_ index (thin contours and color shading) on the background of the climatological jets (black arrows). In the Euro-Atlantic sector, the GPH anomalies form a dipole. During the positive polarity of the SAT_A_ index, this dipole consists of an Arctic trough (lobe of negative GPH anomalies) and a ridge (lobe of positive GPH anomalies) in middle latitudes, with centers of action over Greenland and western Europe, respectively. Since the GPH gradients drive geostrophic winds, the dipole represents cyclonic motion in the north (the reinforced upper-tropospheric polar vortex around Greenland) and anticyclonic motion in the south. These upper-tropospheric vortices correspond to the enhanced Icelandic Low and Azores High at the surface (Fig. 2a). The meridional GPH gradients across their common rim drive anomalous westerlies (see the arrows in Fig. 2b for the pattern of significant wind anomalies at 300 hPa regressed onto the related SLP_EA_ index). These anomalous westerlies reflect the poleward migration of the North Atlantic jet. In Fig. 2c, this migration is recognised by a more southern location of the climatological jet than the boundary between the anomalous vortices.

The upper-tropospheric wind anomalies in the Euro-Atlantic region are accompanied by an anomalous vortex over Asia with the center of action above Lake Baikal. In the positive phase of the SAT_A_/SLP_EA_ index, this “Lake Baikal” vortex is anticyclonic and corresponds to a well-pronounced ridge straddling the climatological Eurasian polar front jet (Fig. 2b, arrows, and Fig. 2c). While the vortices in the Euro-Atlantic region are largely equivalent barotropic (anomalous winds near the surface tend to keep the direction of strong wind anomalies in the upper troposphere), the “Lake Baikal” vortex has a more baroclinic structure. It considerably weakens and migrates southward in the lower troposphere. At the surface, its remnants can be recognised as weak SLP anomalies in southern Asia (Fig. 2a,b). A baroclinic structure of anomalous vortices over eastern Eurasia was also noted in the context of intraseasonal amplification of the Siberian High^42^ and intraseasonal variation of the strength of the East Asian trough^43^.

Following the related study^8^, the “Lake Baikal” vortex is characterised by the GPH_LB_ index defined as standardised anomalies of *Z*_300_ averaged around Lake Baikal (LB box in Fig. 2c). This index correlates quite highly with the NAO index ($r=0.71$) and even higher with the SLP_EA_ index ($r=0.84$; see Supplementary Fig. S1c for comparison of the time series) and the SAT_A_ index ($r=0.89$; see Table 1). These high correlations suggest that the “Lake Baikal” vortex controls thermal variability underneath and, consequently, contributes hand in hand with the anomalous surface westerlies in the Euro-Atlantic sector to coherent wintertime air temperature variations at the ground across the entire Eurasian continent. Such a scenario is consistent with the anomaly patterns of SAT and surface wind regressed onto the GPH_LB_ index (Fig. 2d), which strongly resemble the corresponding NAO-covariant patterns (Fig. 1b). The anomaly pattern of SAT regressed onto the GPH_LB_ index after using regression to remove the signal associated with the NAO index exhibits a significant lobe in northern Asia but not in either Europe or Greenland (Supplementary Fig. S2a). Conversely, the anomaly pattern of SAT regressed onto the NAO index after removing the GPH_LB_-covariant signal from the time series shows the canonical Europe-Greenland seesaw in temperature anomalies but lacks their Asian lobe (Supplementary Fig. S2b), further supporting the scenario that the Asian lobe is controlled by the “Lake Baikal” vortex.

### Vertical structure of temperature anomalies over Asia

It was previously suggested from an analysis of downstream NAO influences on subseasonal timescales^40^ that upper-tropospheric circulation anomalies in the Lake Baikal area may drive air temperature variations in northern Asia via surface-reaching displacements of isentropic surfaces. This mechanism should be even more relevant on the seasonal timescale. Indeed, the link between the anomalies of the winter mean SAT and upper-tropospheric circulation over northern Asia is much stronger than the corresponding link between the month-to-month anomalies in winter. While the correlations of the SAT_A_ index with the local GPH anomalies at 300 hPa in the Lake Baikal area exceed 0.8 for the winter mean data, the corresponding correlations drop below 0.4 for the month-to-month anomalies (Supplementary Fig. S3a).

**Figure 4:**
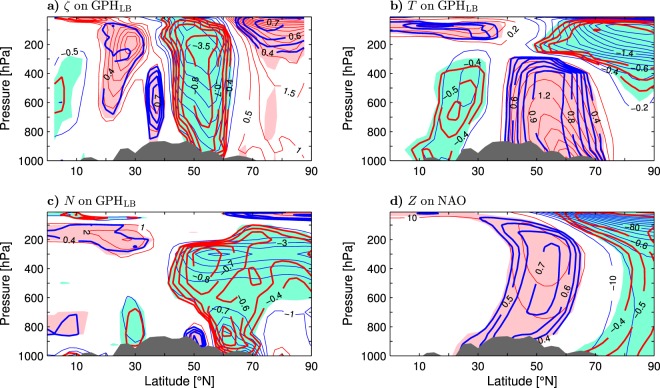
Latitude-pressure cross-section of wintertime atmospheric anomalies averaged between 90° and 125°E and regressed onto indices of Eurasian climate variability in the ESO period. (**a**–**c**) Anomalies of the relative vorticity $\zeta$, temperature *T* and buoyancy frequency *N* (thin contours and color shading), respectively, regressed onto the GPH_LB_ index defined as standardised anomalies of the geopotential height at 300 hPa averaged over the Lake Baikal area (LB box in Fig. 2c). The CI is 0.5 × 10^−6^ s^−1^, 0.2 K and 1 × 10^−4^ s^−1^ per unit GPH_LB_ index, respectively. Thick contours are the correlation coefficients (only contours of $|r|\ge 0.4$ are plotted). Dark shading shows approximate orography at 107.5°E. The thin contour and other shading colors are explained in the caption to Fig. 1. (**d**) as (**a**–**c**) but for the anomalies of the geopotential height regressed onto the NAO index. The CI is 10 gpm per unit NAO index.

To further support the scenario of an upper-tropospheric regulation of the wintertime SAT variability in northern Asia, Fig. 4a–c displays the latitude-pressure distributions of the GPH_LB_-covariant anomalies of the relative vorticity $\zeta$, air temperature *T* and buoyancy frequency *N* averaged from 90° to 125°E (thin contours and color shading) together with the corresponding correlations (thick contours). In the Lake Baikal area, the GPH_LB_-covariant anomalies of the relative (and hence absolute) vorticity extend throughout the atmosphere but have the largest amplitude in the upper troposphere/lower stratosphere (Fig. 4a). During the anticyclonic polarity of the “Lake Baikal” vortex (positive GPH anomalies and negative anomalies of $\zeta$), anomalously cold temperatures in the polar lower stratosphere coexist with anomalously warm lower-stratospheric temperatures in low latitudes and, therefore, strengthen the north-to-south temperature gradient above the tropopause (Fig. 4b). At the same time, the thermal contrast between the low and moderately-high latitudes is weakened below the tropopause, as indicated by a tropospheric dipole of temperature anomalies that is out-of-phase with the dipole in the lower stratosphere. Significant anomalies in the tropospheric lobes reach the surface. In contrast to the southern lobe where the largest tropospheric temperature anomalies appear at upper levels, in the northern lobe, the largest temperature anomalies are found at the surface, on the northern edge of the “Lake Baikal” vortex. However, the temperature anomalies within the vortex (between 50° and 60°N) are related to the GPH_LB_ index very tightly throughout the troposphere, with correlations exceeding 0.95 between 850 and 400 hPa. These anomalies change sign at the core level (300–250 hPa) of the vortex (compare the thin contours in Fig. 4a,b). In the positive phase of the GPH_LB_ index, such a structure of temperature anomalies corresponds to decreased static stability throughout the troposphere, with an extreme magnitude at 300 hPa (Fig. 4c). Therefore, the anomalous anticyclonic absolute vorticity and reduced static stability in the “Lake Baikal” vortex are manifestations of a negative upper-tropospheric potential vorticity anomaly. Such an anomaly can produce surface warming by pushing isentropic surfaces downward^44^. Conversely, in the negative phase of the GPH_LB_ index, a positive upper-tropospheric potential vorticity anomaly corresponding to the anomalous cyclonic absolute vorticity and increased static stability in the “Lake Baikal” vortex can produce surface cooling by pulling isentropic surfaces upward. While these processes do not require teleconnections to the North Atlantic region (see the discussion of Supplementary Fig. S2a above), they are often associated with such teleconnections, as indicated by significant NAO-covariant upper-tropospheric GPH anomalies in the Lake Baikal area (Fig. 4d).

**Figure 5:**
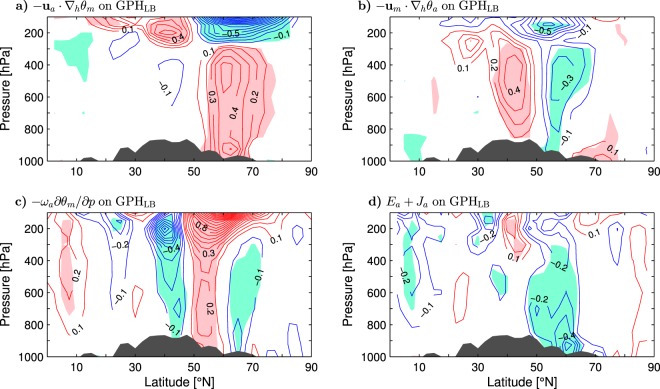
Latitude-pressure cross-section of wintertime anomalies of the terms in the thermodynamic equation averaged between 90° and 125°E and regressed onto the GPH_LB_ index defined as standardised anomalies of the geopotential height at 300 hPa averaged over the Lake Baikal area (LB box in Fig. 2c) in the ESO period. (**a**) Mean temperature advection by the anomalous horizontal wind. (**b**) Anomalous temperature advection by the mean horizontal wind. (**c**) Mean temperature advection by the anomalous vertical motion. (**d**) Anomalous eddy heat flux convergence and diabatic heating. In (**a**–**d**) the CI is 0.1 K day^−1^ per unit GPH_LB_ index. Dark shading shows approximate orography at 107.5°E. The thin contour and other shading colors are explained in the caption to Fig. 1.

To explain the surface amplification of temperature anomalies in the “Lake Baikal” vortex form the perspective of the heat balance, Fig. 5 shows the latitude-pressure distribution of the GPH_LB_-covariant terms of the thermodynamic equation averaged zonally over the 90°–125°E longitudes (only anomalies in the layer 1000–100 hPa are plotted). On the northern side of the vortex, the tropospheric and lower-stratospheric temperature anomalies are maintained by mean temperature advection by the anomalous wind, as indicated by the sign of the respective lobes in the distribution of $-{{\bf{u}}}_{a}\cdot {\nabla }_{h}{\theta }_{m}$ (Fig. 5a). This advection has nearly the same magnitude throughout its tropospheric lobe. Anomalous temperature advection by the mean wind displaces the tropospheric temperature anomalies towards the southern side of the vortex, as indicated by a tropospheric dipole in the distribution of $-{{\bf{u}}}_{m}\cdot {\nabla }_{h}{\theta }_{a}$ (Fig. 5b). This dipole is confined to the free troposphere so that the total anomalous horizontal advection ($-{{\bf{u}}}_{a}\cdot {\nabla }_{h}{\theta }_{m}-{{\bf{u}}}_{a}\cdot {\nabla }_{h}{\theta }_{m}$; the nonlinear contribution $-{{\bf{u}}}_{a}\cdot {\nabla }_{h}{\theta }_{a}$ is negligible) exhibits a maximum at the surface on the northern side of the vortex. This maximum maintains the corresponding temperature maximum (Fig. 4b). Anomalous temperature advection by the total (mean plus anomalous) pressure velocity is negligible (not shown) while mean temperature advection by the anomalous pressure velocity ($-{\omega }_{a}\partial {\theta }_{m}/\partial p$) maintains tropospheric temperature anomalies around the central latitude of the vortex and destroys them on the southern and northern sides of the vortex (Fig. 5c). In the lower stratosphere, mean temperature advection by the anomalous pressure velocity counteracts mean temperature advection by the anomalous wind.

The combined forcing by anomalous eddy heat flux convergence and diabatic heating (${E}_{a}+{J}_{a}$) tends to destroy the temperature anomalies in the “Lake Baikal” vortex throughout the troposphere. During the anticyclonic phase of the GPH_LB_ index, this forcing represents a heat sink having an extreme magnitude at or near the surface (Fig. 5d). A localised heat sink should produce positive potential vorticity above the sink that is apportioned between increased absolute vorticity (cyclonic circulation) and increased static stability^45^. Such changes are of opposite sign to the changes of absolute vorticity and static stability associated with the GPH_LB_ index (Fig. 4a,c). Therefore, the ${E}_{a}+{J}_{a}$ forcing should also exert negative dynamic feedback on the “Lake Baikal” vortex. Two-way interactions between upper-tropospheric circulation anomalies and near-surface processes also contribute to intraseasonal climate variations over Asia^42,43^.

## Relation to Variations in Storm Track Activity

### Displacements of the North Atlantic storm track

**Figure 6:**
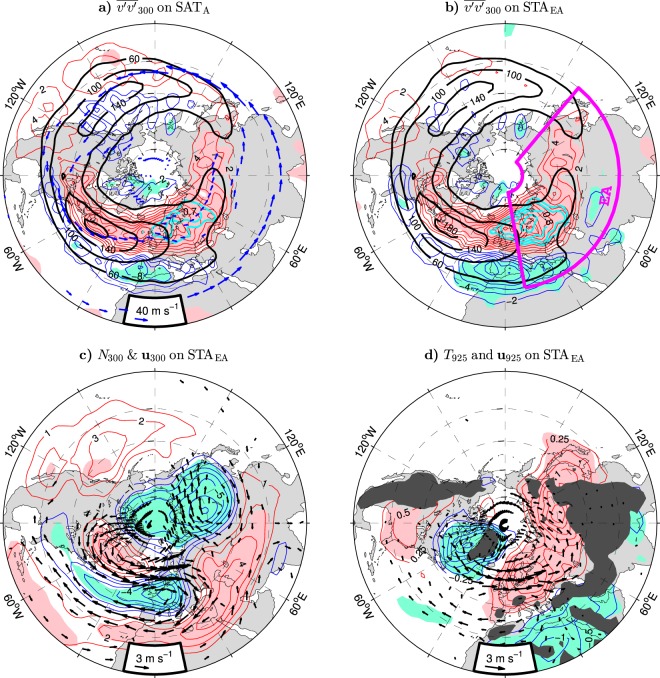
Wintertime storm track activity variations and other atmospheric anomalies in the NH extratropics associated with indices of Eurasian climate variability in the ESO period. (**a** and **b**) Anomalies of ${\overline{v^{\prime} v^{\prime} }}_{300}$ (thin contours and color shading) regressed onto the SAT_A_ and STA_EA_ indices, respectively. The CI is 2 m^2^ s^−2^ per unit index. Thick black contours show the wintertime climatology of ${\overline{v^{\prime} v^{\prime} }}_{300}$ (in m^2^ s^−2^). Thick cyan contours are the isolines of high correlations ($|r|=\{0.6,0.7,0.8,0.9\}$). In (**a**) blue arrows depict the wintertime climatology of jet streams. (**c** and **d**) STA_EA_-covariant anomalies (thin contours and color shading) of the buoyancy frequency at 300 hPa and surface air temperature at 925 hPa, respectively. The CI 1 × 10^−4^ s^−1^ and 0.25 K per unit STA_EA_ index, respectively. Arrows show the STA_EA_-covariant anomalies of the horizontal wind velocity (in m s^−1^ per unit STA_EA_ index, subsampled and masked if both components are nonsignificant at the 95% confidence level) at 300 and 925 hPa, respectively. In (**d**) dark shading masks approximately areas where the climatological pressure at the surface is lower than 925 hPa. The thin contour and other shading colors used in (**a**–**d**) are explained in the caption to Fig. 1. The SAT_A_ and STA_EA_ indices are defined in the caption to Table 1. The maps were generated by MathWorks MATLAB R2014a with M_Map (http://www.eoas.ubc.ca/~rich/map.html).

Higher-than-normal air temperatures over northern Asia are related to an intensification, northward migration and eastward expansion of the North Atlantic storm track. These features are shown in Fig. 6a displaying the anomaly pattern of STA defined as the wintertime variance of the high-pass filtered daily meridional wind at 300 hPa (${\overline{v^{\prime} v^{\prime} }}_{300}$, see Methods) regressed onto the SAT_A_ index (thin contours and color shading) on the background of the wintertime climatology of ${\overline{v^{\prime} v^{\prime} }}_{300}$ in the ESO period (thick black contours). The pattern of the SAT_A_-covariant anomalies of ${\overline{v^{\prime} v^{\prime} }}_{300}$ closely resembles the corresponding pattern associated with the STA_EA_ index. The STA_EA_ index is defined as the PC time series of the leading EOF mode of the variability in ${\overline{v^{\prime} v^{\prime} }}_{300}$ over extratropical Eurasia (EA box in Fig. 6b). Both patterns (see the thin contours and color shading in Fig. 6b for the STA_EA_-covariant anomalies of ${\overline{v^{\prime} v^{\prime} }}_{300}$) exhibit a zonally-elongated lobe of significant STA anomalies north of about 45° that extends from eastern North America across the North Atlantic and Europe to Asia and, over eastern Europe, from the Black Sea to the Barents Sea. This northern lobe is accompanied by a southern lobe of weaker and far less significant out-of-phase STA anomalies with the center of action over the Iberian Peninsula. In the northern lobe, the largest anomalies appear over the North Sea region, but the most significant anomalies are found slightly downstream, over northern Europe at about 60°N (see the thick cyan contours in Fig. 6a,b for the isolines of high correlations). This feature underscores the importance of processes that tend to extend the North Atlantic storm track along the Eurasian jet stream (see the arrows in Fig. 6a for the NH jets climatology) for climate variability in northern Asia.

The STA_EA_ index correlates highly with the SAT_A_ index ($r=0.80$; see Table 1) and the SLP_EA_ index ($r=0.88$; see Supplementary Fig. S1d for comparison of the time series), as well as the NAO ($r=0.83$) and GPH_LB_ ($r=0.76$) indices^8^. Consistent with these tight relations, the STA_EA_-covariant anomaly patterns exhibit all major features associated with the other indices, including the upper-tropospheric “Lake Baikal” vortex and large static stability anomalies in the vortex area (see Fig. 6c; arrows for the wind anomalies and thin contours and color shading for the buoyancy frequency anomalies at 300 hPa). They show the equivalent barotropic structure of the zonal wind anomalies in the Euro-Atlantic sector (compare the arrows in Fig. 6c with the arrows in Fig. 6d representing the wind anomalies at 925 hPa). They also exhibit all NAO-like thermal features in the lower troposphere, including the European and Asian cores in the broad lobe of air temperature anomalies over northern Eurasia (see the thin contours and color shading in Fig. 6d for the temperature anomalies at 925 hPa). Taken together, the anomaly patterns in Fig. 6 strongly suggest that the displacements of the North Atlantic storm track play a key role in the tight relation of air temperatures over Eurasia to the large-scale circulation anomalies. This scenario is further supported by a much stronger link of air temperatures in northern Asia to the STA and wind anomalies in the Euro-Atlantic sector for the winter mean data than the month-to-month anomalies. Indeed, whereas the correlations of the SAT_A_ index with the anomalies of ${\overline{v^{\prime} v^{\prime} }}_{300}$ over Europe exceed 0.7 for the winter mean data, the corresponding correlations drop below 0.3 for the month-to-month anomalies (Supplementary Fig. S3b). Similarly, the winter mean and month-to-month SAT_A_ indices correlate with the corresponding upper-tropospheric vortices in the Euro-Atlantic region at levels above 0.7 and below 0.3, respectively (Supplementary Fig. S3a).

### Synoptic eddy forcing

**Figure 7:**
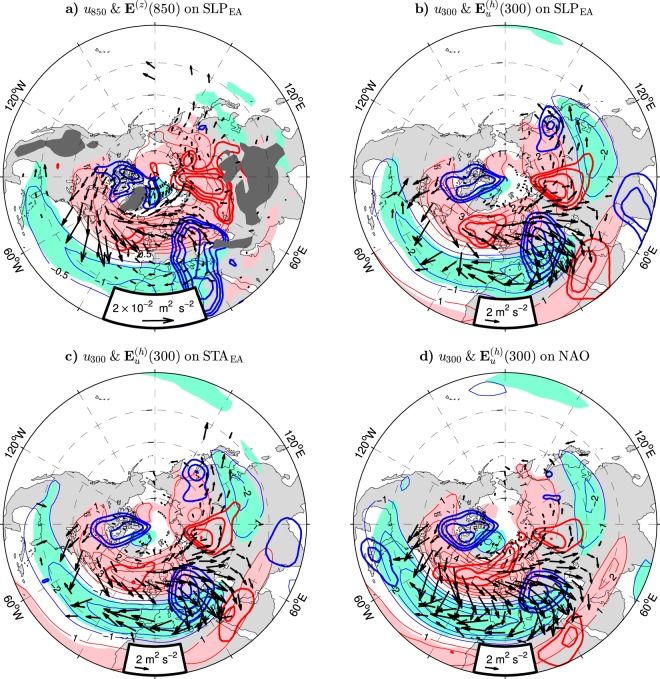
Wintertime synoptic eddy forcing and wind anomalies in the NH extratropics associated with indices of Eurasian climate variability in the ESO period. (**a**) Anomalies of the zonal wind velocity (positive eastward; thin contours and color shading) and the ${{\bf{E}}}^{(z)}$ vector (arrows) at 850 hPa regressed onto the SLP_EA_ index. The CI for *u*_850_ is 0.5 m s^−1^ per unit SLP_EA_ index. Thick contours are the correlation coefficients (only contours of $|r|\ge 0.4$ are plotted) of the meridional wind anomalies (positive northward) at 850 hPa with the SLP_EA_ index. The zonal and meridional components of ${{\bf{E}}}^{(z)}$ are defined in Eqs 6 and 7, respectively. (**b**) As (**a**) but for the regressions of the zonal wind velocity and the ${{\bf{E}}}_{u}^{(h)}$ vector defined in Eq. 6 and the correlations of the meridional wind anomalies at 300 hPa. The CI for *u*_300_ is 1 m s^−1^ per unit SLP_EA_ index. (**c** and **d**) As (**b**) but for the regressions and correlations with the STA_EA_ and NAO indices, respectively. In (**a**–**d**), the vectors are in m^2^ s^-2^ per unit index, subsampled and masked if both components are nonsignificant at the 95% confidence level. The SLP_EA_, STA_EA_ and NAO indices are defined in the caption to Table 1. In (**a**) dark shading masks approximately areas where the climatological pressure at the surface is lower than 850 hPa. The maps were generated by MathWorks MATLAB R2014a with M_Map (http://www.eoas.ubc.ca/~rich/map.html).

Transient eddy forcing of the mean flow can be expressed by the extended Eliassen-Palm vectors **E**_*u*_ and **E**_*v*_ (see Eqs 6 and 7 in Methods). Divergences of these vectors represent local eddy-induced accelerations of the zonal and meridional winds, respectively^46^. In the lower troposphere, these accelerations occur mainly through baroclinic processes described by the vertical components ${E}_{u}^{(z)}$ and ${E}_{v}^{(z)}$ of the **E**_*u*_ and **E**_*v*_ vectors. The vertical gradient of ${E}_{u}^{(z)}\propto \overline{v^{\prime} T^{\prime} }$, where $\overline{v^{\prime} T^{\prime} }$ is the poleward eddy heat flux, represents the baroclinic eddy forcing of the mean zonal flow. The corresponding forcing of the mean meridional flow is determined by the vertical gradient of ${E}_{v}^{(z)}\propto -\,\overline{u^{\prime} T^{\prime} }$, where $\overline{u^{\prime} T^{\prime} }$ is the eastward eddy heat flux. Assuming that the eddy heat flux vanishes at the surface, a positive anomaly of the poleward (resp. eastward) eddy heat flux at a lower-tropospheric level should drive a westerly (resp. northerly) wind anomaly at that level. The relation between the winter mean wind anomalies and their baroclinic forcing due to synoptic eddies at 850 hPa associated with the leading mode of variability in the surface circulation over Eurasia is summarised in Fig. 7a, in which the arrows show the anomaly pattern of significant SLP_EA_-covariant vectors ${{\bf{E}}}^{(z)}=({E}_{u}^{(z)},{E}_{v}^{(z)})$. These vectors are plotted on the background of the corresponding anomalies of the zonal wind *u* (thin contours and shading) and the correlations of the meridional wind *v* with the SLP_EA_ index (thick contours). During the positive phase of this index, the enhanced westerlies over the northern North Atlantic and adjacent lands, including most of Europe, coincide with increased poleward eddy heat flux in this region. Similarly, the weakened westerlies in the area extending from the Mediterranean Sea to the Gulf of Mexico coincide with reduced poleward eddy heat flux in this area. Therefore, the zonal wind anomalies in the Euro-Atlantic sector are driven or maintained by the synoptic eddies. The anomalous zonal eddy heat flux supports meridional wind anomalies mainly over central Europe and the Canadian Arctic Archipelago where anomalous northerlies coincide with eastward eddy heat flux anomalies.

In the upper troposphere, transient eddy forcing of the mean flow occurs mainly through barotropic processes described by the horizontal components ${{\bf{E}}}_{u}^{(h)}$ and ${{\bf{E}}}_{v}^{(h)}$ of the **E**_*u*_ and **E**_*v*_ vectors. The poleward component of ${{\bf{E}}}_{u}^{(h)}$ and the eastward component of ${{\bf{E}}}_{v}^{(h)}$ represent the equatorward eddy momentum flux ($-\overline{u^{\prime} v^{\prime} }$). More generally, the ${{\bf{E}}}_{u}^{(h)}$ and ${{\bf{E}}}_{v}^{(h)}$ vectors are interrelated in such a way that the divergence and the curl of one of them (e.g., ${\nabla }_{h}\cdot {{\bf{E}}}_{u}^{(h)}$ and ${\bf{k}}\cdot {\nabla }_{h}\times {{\bf{E}}}_{u}^{(h)}\equiv {\nabla }_{h}\cdot {{\bf{E}}}_{v}^{(h)}$) provide full information on the barotropic eddy forcing of both wind components. Arrows in Fig. 7b show the anomaly pattern of the SLP_EA_-covariant ${{\bf{E}}}_{u}^{(h)}$ vectors due to synoptic eddies at 300 hPa on the background of the corresponding anomalies of the zonal wind (*u*_300_; thin contours and shading) and the correlations of the meridional wind at the same level (*v*_300_) with the SLP_EA_ index (thick contours). Major anomalies of ${{\bf{E}}}_{u}^{(h)}$ appear in the Euro-Atlantic sector. During the positive phase of the SLP_EA_ index, they diverge in the subpolar region and converge at lower latitudes. These divergences and convergences should accelerate westerly winds in the north and decelerate them in the south. They indeed correspond to the SLP_EA_-covariant strengthening of the upper-tropospheric westerlies at 50°–70°N and their weakening at 30°–40°N. Moreover, the anomalous ${{\bf{E}}}_{u}^{(h)}$ vectors make a vigorous loop over Europe which should drive (through the curl of ${{\bf{E}}}_{u}^{(h)}$) meridional wind anomalies in this area. Northerly wind anomalies over Europe at 15°–45°E are indeed observed during the positive polarity of the SLP_EA_ index when the anomalous ${{\bf{E}}}_{u}^{(h)}$ vectors are anticyclonic in this area.

The anomaly pattern of the upper-tropospheric ${{\bf{E}}}_{u}^{(h)}$ vectors associated with the SLP_EA_ mode (Fig. 7b) mirrors the corresponding pattern associated with the leading mode of storm track activity variations over Eurasia (Fig. 7c), reflecting the already noted quasi-equivalence of these modes. In the Euro-Atlantic sector, both patterns are similar to the corresponding pattern of the ${{\bf{E}}}_{u}^{(h)}$ vectors associated with the month-to-month AO mode derived from a numerical model^35^. However, in contrast to the latter, they show a significant propagation of eddy activity from Scandinavia into Asia and lack a significant connection between the Pacific and Atlantic storm tracks across North America. Still, the link of the winter mean upper-tropospheric circulation anomalies over Asia to the synoptic eddy forcing in the ESO period should mainly reflect nonlocal connections. The anomalous ${{\bf{E}}}_{u}^{(h)}$ vectors associated with the SLP_EA_/STA_EA_ mode are less significant over Asia than Europe and, over Asia, they do not exhibit a clear pattern able to explain the “Lake Baikal” vortex (Fig. 7b,c). Moreover, the NAO-covariant ${{\bf{E}}}_{u}^{(h)}$ vectors are generally not significant over Asia (Fig. 7d) despite the significant relation of the “Lake Baikal” vortex to the NAO (Fig. 4d).

## Relation to Variations in Stationary Planetary Waves and Zonal Mean Winds

### Anomalous wavenumber-3 pattern

**Figure 8:**
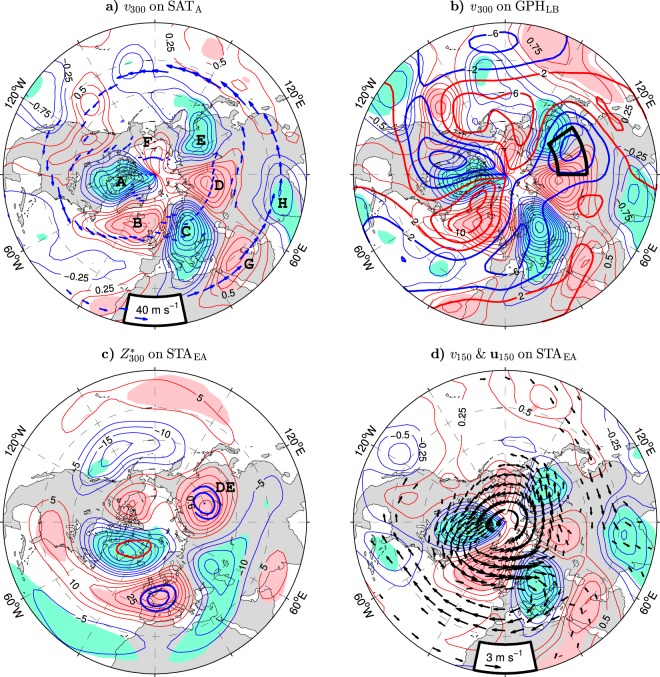
Wintertime upper-tropospheric and lower-stratospheric anomalies in the NH extratropics associated with indices of Eurasian climate variability in the ESO period. (**a** and **b**) Anomalies of the meridional wind velocity at 300 hPa (positive northward; thin contours and color shading) regressed onto the SAT_A_ and GPH_LB_ indices, respectively. The CI is 0.25 m s^−1^ per unit index. In (**a**) blue arrows depict the wintertime climatology of jet streams. Letters A-H mark lobes of the *v*_300_ anomalies discussed in the text. In (**b**) thick contours show the wintertime climatology of *v*_300_ (in m s^−1^). (**c**) Anomalies of the zonally asymmetric component of the geopotential height at 300 hPa (${Z}_{300}^{\ast }$, thin contours and color shading) regressed onto the STA_EA_ index. The CI is 5 gpm per unit STA_EA_ index. Thick contours are the isolines of high correlations ($|r|=\{0.6,0.7,0.8\}$) between ${Z}_{300}^{\ast }$ and the STA_EA_ index. Letters DE mark the lobe of ${Z}_{300}^{\ast }$ anomalies corresponding to the “Lake Baikal” vortex. (**d**) STA_EA_-covariant anomalies of the horizontal wind velocity (arrows; in m s^−1^ per unit index, subsampled and masked if both components are nonsignificant at the 95% confidence level) and its meridional component (positive northward; thin contours and color shading; CI: 0.25 m s^−1^ per unit index) at 150 hPa. The thin contour and shading colors used in (**a**–**d**) are explained in the caption to Fig. 1. The SAT_A_, GPH_LB_ and STA_EA_ indices are defined in the caption to Table 1. The maps were generated by MathWorks MATLAB R2014a with M_Map (http://www.eoas.ubc.ca/~rich/map.html).

Stationary planetary waves are driven by zonally-asymmetric forcings, such as land orography, sources/sinks of diabatic heating, and transient eddies^47,48^. Therefore, changes in the eddy momentum and heat fluxes by storm track displacements as well as the associated reorganisation of diabatic heating sources or interactions of the induced circulation anomalies with land orography may produce anomalous planetary waves, promoting non-local responses. Anomaly patterns of the meridional wind at 300 hPa indicate that quasi-stationary planetary waves are important for connecting the upper-tropospheric winds and lower-tropospheric temperatures over Asia to the circulation and storm track anomalies in the Euro-Atlantic region. The anomalies of *v*_300_ regressed onto the SAT_A_ and GPH_LB_ indices (see the thin contours and color shading in Fig. 8a,b) exhibit a quasi-zonal wavenumber-3 circumglobal structure in high latitudes (approximately between 50°N and 75°N). A similar structure is associated with the SLP_EA_, STA_EA_, and NAO indices (see the thick contours in Fig. 7b–d). The six lobes of this high-latitude CWP3 are denoted by letters A-F in Fig. 8a. Lobe A (over the Canadian Arctic Archipelago) and lobe B (over the northern North Atlantic) correspond to, respectively, the western and eastern limbs of the anomalous Greenland vortex (Fig. 2b,c). Lobes D and E over northern Asia correspond to, respectively, the western and eastern limbs of the “Lake Baikal” vortex (marked by the black box in Fig. 8b). Lobe D is separated from lobe B by a strong lobe located over central Europe (lobe C). Lobe E is separated from lobe A by a weak and much less significant lobe F that is confined to the high Arctic (to the area north of the Bering Strait). Lobes C-E are aligned with the climatological Eurasian polar front jet (see the arrows in Fig. 8a). In the south, they are accompanied by lobes (marked as G and H in Fig. 8a) that are located on the climatological axis of the northern Africa-southern Asia subtropical jet. These lobes seem to be part of a less significant low-latitude wavetrain having a distorted wavenumber-5 structure. Their relation to transient eddies in the Euro-Atlantic region is consistent with a recent analysis of month-to-month circulation variability along the wintertime subtropical Asian jet^49^.

The anomalous high-latitude CWP3 represents a shift of a quasi-zonal wavenumber-3 climatological planetary wave. This shift is shown in Fig. 8b by superimposing the climatological meridional wind at 300 hPa (thick contours) on the GPH_LB_-covariant anomalies of this wind. The shift has zonal and meridional components. The anomalous wavetrain appears on the average about 10° of latitude poleward of the climatological one. In the zonal direction, it is approximately in quadrature with the climatological wavetrain. In particular, the Asian lobes (D and E) of the anomalous wavetrain straddle one of the three strongest climatological lobes, that is, the one co-located with the center of the “Lake Baikal” vortex.

While the circumglobal character of the high-latitude wavetrain is well pronounced in the meridional wind anomalies, it is less evident in the corresponding anomalies of the geopotential height (Fig. 2c) since, because of the geostrophic balance, *v* is proportional to the zonal gradient of *Z* and thus has almost no zonal mean. In contrast, the geopotential height anomalies include a significant zonally-symmetric component corresponding to the anomalies of the zonal-mean zonal wind {*u*} discussed below. This component somewhat masks the high-latitude wavetrain that is present in the zonally-asymmetric component Z* of the geopotential height anomalies (see the thin contours and color shading in Fig. 8c for the STA_EA_-covariant anomalies of Z* at 300 hPa). From all lobes of the ${Z}_{300}^{\ast }$ wavetrain, the Asian one (marked as DE in Fig. 8c) is linked most significantly to the STA_EA_ index. This feature is shown by the isolines of high correlations of the anomalies of ${Z}_{300}^{\ast }$ with the STA_EA_ index in Fig. 8c (thick contours). The highest correlation (0.80) is found in the center of the Asian lobe located north of Lake Baikal at 60°N, supporting the scenario that the “Lake Baikal” vortex is linked to the storm track variability in the Euro-Atlantic sector via planetary waves.

**Figure 9:**
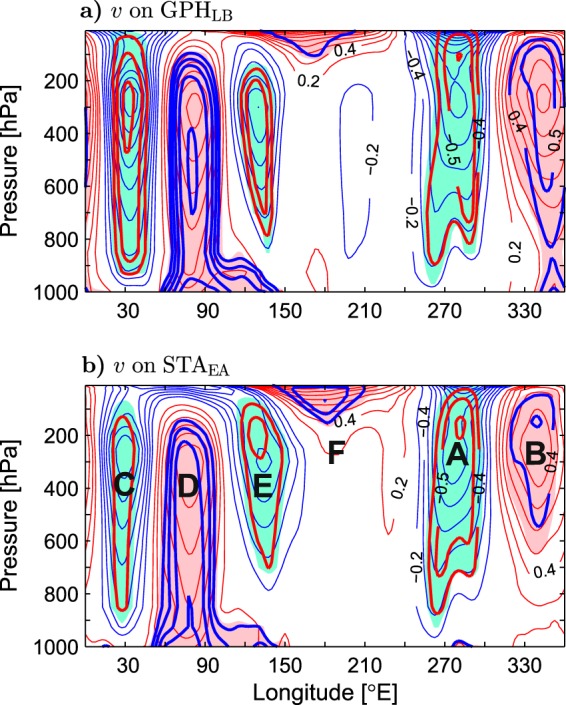
Longitude-pressure cross-section of wintertime anomalies of the meridional wind averaged between 50° and 70°N associated with indices of Eurasian climate variability in the ESO period. (**a** and **b**) Anomalies of *v* (positive northward; thin contours and color shading) regressed onto, respectively, the GPH_LB_ and STA_EA_ indices defined in the caption to Table 1. The CI is 0.2 m s^−1^ per unit index. The thin contour and shading colors are explained in the caption to Fig. 1. Thick contours are the correlation coefficients (only contours of $|r|\ge 0.4$ are plotted). Letters A-F mark anomalous lobes discussed in the text.

The vertical structure of the high-latitude CWP3 is illustrated in Fig. 9a,b showing the longitude-pressure distributions of the meridional wind anomalies (thin contours and color shading) averaged from 50° to 70°N and regressed onto the GPH_LB_ and STA_EA_ indices, respectively, together with the corresponding correlations (thick contours). Generally, the CWP3 wavetrain has an equivalent barotropic structure in the free troposphere, below the cores of the meridional velocity anomalies at 300–250 hPa. An exception is lobe F, which is a stratospheric feature. A significant lower-stratospheric signature (the highest correlations at 200–100 hPa) is also found in the Greenland vortex lobes (A and B). From the perspective of the surface climate variability, the most outstanding lobe is the one over northwestern Asia (lobe D). This lobe has only a weak signature in the lower stratosphere but reaches the surface. In fact, it is most significant just at the surface where it correlates very highly ($r=0.86$) with the GPH_LB_ index and also highly ($r=0.76$) with the STA_EA_ index. Moreover, at the surface, lobe D spreads significantly eastward underneath lobe E of the upper-tropospheric wavetrain. Therefore, in agreement with the patterns of surface and near-surface wind anomalies (Figs 2d and 6d), most of northern Asia is under the influence of anomalous meridional winds that strongly affect local air temperatures (Fig. 3a–c).

### Anomalous zonal-mean zonal winds

**Figure 10:**
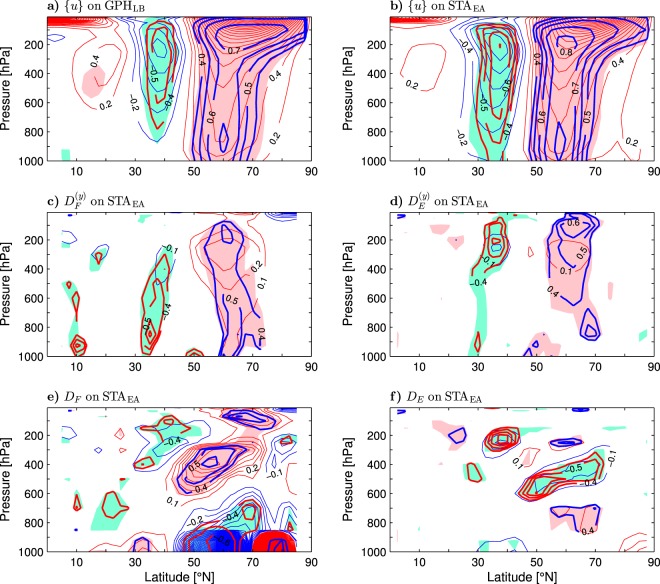
Latitude-pressure cross-section of wintertime anomalies of the zonal-mean zonal wind and divergence of wave activity fluxes associated with indices of Eurasian climate variability in the ESO period. (**a** and **b**) Anomalies of {*u*} (positive eastward; thin contours and color shading) regressed onto, respectively, the GPH_LB_ and STA_EA_ indices defined in the caption to Table 1. The CI is 0.2 m s^−1^ per unit index. The thin contour and shading colors are explained in the caption to Fig. 1. Thick contours are the correlation coefficients (only contours of $|r|\ge 0.4$ are plotted). (**c** and **d**) As (**b**) but for the anomalies of the meridional divergence of the quasi-stationary (${D}_{F}^{(y)}$) and synoptic (${D}_{E}^{(y)}$) wave activity fluxes, respectiv**e**ly. (**e** and **f**) As (**b**) but for the anomalies of the total divergence of the quasi-stationary (*D*_*F*_) and synoptic (*D*_*E*_) wave activity fluxes, respectively. In (**c**–**f**) the CI is 0.1 m s^−1^ day^−1^ per unit STA_EA_ index. See Eqs 5 and 8 in Methods for the definition of *D*_*F*_, ${D}_{F}^{(y)}$, *D*_*E*_ and ${D}_{E}^{(y)}$.

In the lower stratosphere, the six lobes of the high-latitude CWP3 are anchored to an anomalous quasi-annular flow corresponding, in the positive phase of the GPH_LB_ and STA_EA_ indices, to the strengthening of the cyclonic polar vortex. This feature is illustrated in Fig. 8d displaying the STA_EA_-covariant anomaly patterns of the wind velocity (arrows) and its meridional component (thin contours and color shading) at 150 hPa. These patterns also exhibit significant lower-stratospheric extensions of the Asian lobes of the low-latitude wavetrain in the upper troposphere (lobes G and H in Fig. 8a). During the positive polarity of the STA_EA_ index, these extensions represent a southward diversion of an easterly flow anomaly from the North Pacific (lobe H) and a northward diversion of a westerly subtropical flow from the North Atlantic (lobe G). A cyclonic loop from the latter feeds anomalous easterlies over the North Atlantic found at the latitudes (30°–45°N) of the anomalous easterlies over eastern Asia. Consequently, it contributes to the southern lobe of a dipolar structure of the zonal-mean zonal wind anomalies in the NH extratropics. This structure is demonstrated explicitly in the upper panels of Fig. 10 showing the latitude-pressure distributions of the anomalies of {*u*} associated with the GPH_LB_ and STA_EA_ indices (thin contours and color shading) together with the corresponding correlations (thick contours).

The southern lobe of the anomalous {*u*}-dipole, centered at 37.5°N, has a core at 200 hPa (Fig. 10a,b). The northern lobe exhibits most significant anomalies at about the same level and also near the surface (at 925–850 hPa). However, the correlations within this lobe are quite uniform throughout the troposphere. In the upper troposphere, the lobe is centered at a latitude (60°N) corresponding to the “central“ latitude of the high-latitude CWP3 defined as the average latitude of the centers of action in its five most significant lobes (A-E) at 300 hPa. At any level, the northern lobe of the anomalous {*u*}-dipole is more significant than the southern one. Correlations in the northern lobe reach 0.84 at 60°N and 200 hPa for the STA_EA_ index (Fig. 10b) and 0.76 at 65°N and 150 hPa for the GPH_LB_ index (Fig. 10a). These high correlations and the “co-location” of the high-latitude CWP3 with the northern lobe of the anomalous {*u*}-dipole strongly suggest that the circumglobal teleconnectivity in high latitudes involves feedbacks between synoptic eddies, stationary waves, and zonal-mean zonal wind perturbations.

### Anomalous planetary wave activity fluxes

**Figure 11:**
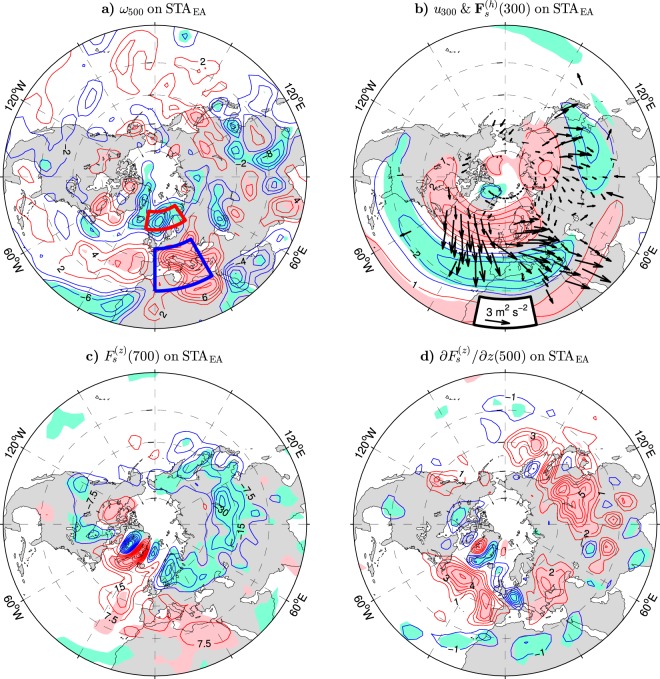
Wintertime tropospheric anomalies in the NH extratropics regressed onto the STA_EA_ index defined as the PC time series of the leading EOF mode of storm track activity variations at 300 hPa over extratropical Eurasia (EA box in Fig. 6b) in the ESO period. (**a**–**d**) Anomalies of the vertical velocity $\omega$ (positive downward) at 500 hPa, zonal velocity *u* (positive eastward) at 300 hPa, vertical component ${F}_{s}^{(z)}$ of the quasi-stationary wave activity flux (positive upward) at 700 hPa and its vertical divergence at 500 hPa, respectively. The CI is 2 × 10^−3^ Pa s^−1^, 1 m s^−1^, 7.5 × 10^−3^ m^2^ s^−2^ and 1 × 10^−6^ m s^−2^ per unit STA_EA_ index, respectively. The contour and shading colors are explained in the caption to Fig. 1. In (**a**) the red and blue boxes delineate areas over which the horizontal wind divergence at 300 hPa is averaged. In (**b**) arrows show anomalies of the horizontal component ${{\bf{F}}}_{s}^{(h)}$ of the quasi-stationary wave activity flux (in m^2^ s^−2^ per unit STA_EA_ index, subsampled and masked if both components are nonsignificant at the 95% confidence level) at 300 hPa. The maps were generated by MathWorks MATLAB R2014a with M_Map (http://www.eoas.ubc.ca/~rich/map.html).

Theoretically, the anticyclonic phase of the “Lake Baikal” vortex could be maintained by eastward propagating quasi-stationary Rossby waves exited by anomalous upper-level wind divergences/convergences in the Euro-Atlantic sector and trapped by the Asian jets waveguide. Regardless of whether such divergences/convergences are driven by the synoptic eddies or vice versa, the steering of the “Lake Baikal” vortex by planetary wave sources in the Euro-Atlantic sector would be consistent with an analysis of downstream NAO influences on subseasonal timescales during warm El-Niño-Southern Oscillation winters^40^. Significant out-of-phase lobes are indeed found in the STA_EA_-covariant anomaly pattern of the vertical velocity $\omega$ at 500 hPa (${\omega }_{500}$; see Fig. 11a). In this pattern, the lobe of negative anomalies of ${\omega }_{500}$ centered over the Nordic Seas corresponds to an anomalous upper-level wind divergence while the lobe of positive anomalies of ${\omega }_{500}$ centered over the Mediterranean Sea corresponds to an anomalous upper-level wind convergence. In the northern region, the wind divergence at 300 hPa averaged over the Norwegian Sea and Scandinavia (red box in Fig. 11a) correlates with the STA_EA_ index at 0.86. The STA_EA_ index is also linked tightly ($r=0.80$) to the wind convergence at 300 hPa averaged over the Mediterranean Sea (blue box in Fig. 11a). Anomalous convergences in this area were suggested to be drivers of local precipitation anomalies^50^ and downstream NAO influences on subseasonal timescales via planetary waves trapped by the subtropical jet^39^.

To examine how the winter mean displacements of the North Atlantic storm track are related to sources and sinks of quasi-stationary Rossby waves, the horizontal (${{\bf{F}}}_{s}^{(h)}$) and vertical (${F}_{s}^{(z)}$) components of the Plumb wave activity flux **F**_*s*_ are calculated (see Eqs 3 and 4 in Methods) and regressed onto the STA_EA_ index. The **F**_*s*_ vector is approximately parallel to the direction of the wave energy propagation^51^. The anomaly pattern of the STA_EA_-related ${{\bf{F}}}_{s}^{(h)}$ vectors at 300 hPa is shown in Fig. 11b (arrows) on the background of the corresponding anomalies of the zonal wind (contours and color shading). In the positive phase of the STA_EA_ index, the anomalous wave activity emanates from the subpolar latitudes of the North Atlantic region where its divergence entails anomalous Rossby wave generation by barotropic processes. This “Atlantic” stream propagates southeastward and piles up wave activity (${{\bf{F}}}_{s}^{(h)}$ anomalies converge) in low latitudes but does not enter into Asia. Over eastern Europe, it confluences with a stream that emanates from the Arctic regions of Asia. This “Asian” stream is most significant in the Lake Baikal area where it propagates mainly equatorward. Its divergence indicates a source of wave activity in the lobe of the westerly wind anomalies on the northern side of the “Lake Baikal” vortex while its convergence indicates a sink of wave activity in the lobe of the easterly wind anomalies on the southern side of the vortex. While differences are found in the Pacific sector, over the Atlantic-Eurasian region, the pattern of the STA_EA_-covariant ${{\bf{F}}}_{s}^{(h)}$ vectors is reminiscent of the corresponding pattern associated with the month-to-month AO variations in a numerical model^35^.

Local sources of anomalous stationary wave activity over Asia are also due to baroclinic processes, as indicated by the vertical component of **F**_*s*_. During the positive phase of the STA_EA_ index, a significant reduction (negative anomaly) of this component occurs at low tropospheric levels over vast areas of northern Eurasia (see Fig. 11c for the regression of ${F}_{s}^{(z)}$ at 700 hPa). Extreme negative anomalies are found in the Lake Baikal area and over Scandinavia. This reduction corresponds to a source of wave activity (divergence of the ${F}_{s}^{(z)}$ anomalies) at upper levels. In the Lake Baikal area, this source is located mainly in the middle troposphere (see Fig. 11d for the regression of the divergence of ${F}_{s}^{(z)}$ at 500 hPa).

The zonally-averaged meridional and vertical components of the **F**_*s*_ vector are proportional to the zonally-averaged equatorward momentum flux {$-{u}^{\ast }{v}^{\ast }$} and poleward heat flux {v*T*} by the stationary waves, respectively. Their divergence *D*_*F*_ (see Eq. 5 in Methods) corresponds to the divergence of the conventional Eliassen-Palm flux, which is a driving agent for the zonal-mean westerlies^52^. An analogous driving agent due to the synoptic eddies (*D*_*E*_) is obtained by zonal averaging of the divergence of the **E**_*u*_ vector (see Eq. 8 in Methods). The latitude-pressure distributions of the STA_EA_-covariant anomalies of *D*_*F*_ and *D*_*E*_ (thin contours and color shading) together with the corresponding correlations (thick contours) are shown in the bottom panels of Fig. 10. The middle panels of this figure display the distributions of the barotropic contribution to these anomalies. The quasi-stationary momentum fluxes (Fig. 10c) conform with the synoptic momentum fluxes (Fig. 10d) in forcing the STA_EA_-covariant meridional displacement of the westerlies (Fig. 10b). At the tropopause level (about 250–300 hPa), the forcing by the quasi-stationary momentum fluxes dominates in the high-latitude lobe of the anomalous westerlies while the forcing by the synoptic momentum fluxes dominates in the mid-latitude lobe. The forcing by the synoptic momentum fluxes tends to be more significant in the lower stratosphere while the forcing by the quasi-stationary momentum fluxes is more significant near the surface. The total forcing by either the quasi-stationary waves (Fig. 10e) or synoptic eddies (Fig. 10f) has a more complicated structure, which is characterised by a vertical tilt in the troposphere imposed by the baroclinic component. Below the tropopause, a weaker forcing by the synoptic heat fluxes generally tends to counteract a stronger forcing by the quasi-stationary heat fluxes. In the lower stratosphere, the quasi-stationary heat fluxes drive the poleward extension of the high-latitude lobe of the anomalous westerlies. At the tropopause, the total forcing by the synoptic eddies is more significant than the total forcing by the quasi-stationary waves not only in the mid-latitude core but also in the high-latitude core of the anomalous westerlies. This feature is not evident in the forcing of the zonal-mean zonal wind anomalies associated with the month-to-month AO variability^35^.

## Discussion

A remarkable feature of climate variability in the North Atlantic-Eurasian region is a robust NAO-related recurrence of a lobe of coherent wintertime air temperature anomalies extending from the Atlantic to the Pacific coast of northern Eurasia. Results from the related study^8^ show that the same wintertime NAO index as employed here accounts for 74% of the variance ($r=0.86$) of the leading mode of the concurrent SAT variability in extratropical Eurasia during the ESO period. Here it is shown that the wintertime SAT anomalies in northern Asia are related to the NAO as strongly as the corresponding SAT anomalies in northern Europe. It is also shown that the SAT_A_ index representing the wintertime area-averaged SAT anomalies in Asia north of 40°N is strongly coupled (83% of the variance explained) to the anomalous surface circulation represented by the leading mode of sea level pressure variability over extratropical Eurasia (the SLP_EA_ mode). The SLP_EA_ mode is an NAO-like mode of variability characterised by anomalous surface westerlies in the Euro-Atlantic region and a northern center of action in the SLP anomaly field moved into the Barents Sea region. A high fraction (79%) of the SAT_A_ variance is also explained by upper-tropospheric circulation anomalies over northern Asia encapsulated in the GPH_LB_ index representing the “Lake Baikal” vortex (an anomalous regional-scale ridge/trough centered over Lake Baikal). Therefore, this vortex and the SLP_EA_ mode of the surface circulation variability are often manifestations of the same large-scale phenomenon. The vertical structure of the GPH_LB_-covariant anomalies of the absolute vorticity and static stability indicates that the “Lake Baikal” vortex regulates the tropospheric temperature variability in northern Asia via displacements of isentropic surfaces in a surface-reaching potential vorticity anomaly. Together with the concurrent heat transport by the anomalous surface westerlies in the Euro-Atlantic sector, it contributes to coherent SAT variations across entire northern Eurasia. When the “Lake Baikal” vortex appears independently from the NAO-related westerly wind anomalies in the Euro-Atlantic sector, it still drives significant SAT anomalies over northern Asia. Without the forcing by the “Lake Baikal” vortex, the NAO-related SAT anomalies do not extend far into Asia. As deduced from other studies^8,40^ and demonstrated here, the impact of the “Lake Baikal” vortex on the SAT variability in northern Asia is much more robust in the case of the winter mean anomalies than the month-to-month anomalies in winter.

It is also found that the linkage of the SAT variability in northern Asia to storm track changes is much more robust for the winter mean than month-to-month anomalies. Moreover, the winter mean SAT anomalies in northern Asia are related more tightly to anomalous upper-tropospheric storm track activity over Europe than Asia. The large-scale anomaly pattern of this activity associated with the SAT_A_ index is reminiscent of the leading mode of storm track activity variations over extratropical Eurasia (the STA_EA_ mode). The latter shares 77% of its variance with the SLP_EA_ mode of the surface circulation variability in that region. All these features suggest that displacements of the North Atlantic storm track play a key role in bridging the surface climate variability in the Euro-Atlantic region and northern Asia. This scenario is also supported by a common wavenumber-3 circumglobal structure of upper-tropospheric/lower-stratospheric anomalies of the meridional wind in high-latitudes associated with the STA_EA_ and GPH_LB_ indices. The anomalous CWP3 wavetrain corresponds to a zonal and meridional shift of an analogous climatological wavetrain. The anomaly patterns of the STA_EA_/SLP_EA_-covariant extended Eliassen-Palm vectors indicate that this shift could be triggered or maintained by the synoptic eddy forcing of the mean flow in the Euro-Atlantic sector. This forcing is not only consistent with the meridional shift of the North Atlantic jet but also supports strong meridional wind anomalies over Europe in the area between two relatively weak lobes of the climatological wavetrain.

The main centers of action in the anomalous CWP3 wavetrain appear in the latitude band of the northern lobe of the STA_EA_/GPH_LB_-covariant extratropical dipole in the zonal-mean zonal wind anomalies. The conventional Eliassen-Palm diagnostics indicate that the synoptic eddies, as well as the quasi-stationary waves, are important drivers of these anomalies. The anomaly patterns of the STA_EA_-covariant Plumb wave activity flux suggest that the linkage of the “Lake Baikal” vortex to the North Atlantic storm track displacements is not maintained via anomalous propagation of quasi-stationary Rossby waves from the North Atlantic region to Asia. These patterns exhibit local barotropic wave activity sources not only in the North Atlantic region but also over northern Asia, where also a strong baroclinic source of wave activity appears at mid-tropospheric levels in the Lake Baikal area.

Conceptually, the relationships between changes in the circulation, storm tracks, and quasi-stationary waves in the Atlantic-Eurasian region summarised above are consistent with the previously demonstrated ability of synoptic eddies to drive NAO-like circulation anomalies in the Euro-Atlantic region^11,53,54^ and zonal-mean zonal wind anomalies associated with the NAO variability^36,55^. They also conform with the ability of quasi-stationary waves to drive the NAO/AO-related storm track variations^14^ and zonal-mean zonal wind anomalies^35,56^. They are also consistent with the ability of the perturbed zonal-mean zonal winds to drive anomalous stationary waves through interactions with the climatological stationary waves^55,57^ and the two-way coupling between the anomalous stationary waves and zonal-mean winds^58^. A novel result, suggested in the related study^8^ and elaborated here, is the likely importance of the anomalous CWP3 wavetrain for the robust relationship between wintertime displacements of the North Atlantic storm track and air temperature anomalies over northern Asia. This feature has remained unnoticed by others probably because the studies on the NAO/AO linkages to planetary waves and storm tracks are usually focused on subseasonal timescales at which relations between climatic anomalies in the Euro-Atlantic sector and northern Asia are not robust. Another reason is that these studies often include data from pre-ESO decades. Before the ESO period, the circulation anomalies over northern Asia might not have been robustly linked to the NAO even on the seasonal timescale^41^.

The vertical structure of the GPH_LB_-covariant anomalies of diabatic heating and transient eddy heat flux convergence indicates that these anomalies exert a local negative dynamic feedback on the “Lake Baikal” vortex. Since only concurrent relations are investigated here, one cannot rule out the scenario in which local anomalies in northern Asia are, at least for some forced events, precursors of large-scale feedbacks leading to the NAO/AO like wintertime circulation changes. Such precursors may be related, for instance, to autumnal snow cover anomalies^59^, which in their turn may depend on Arctic sea ice cover variability^60^. Statistical forecast experiments show that more than 50% of the STA_EA_/NAO mode variability can be predicted from autumnal anomalies of the sea ice extent in the Barents and Kara Seas^8^. The wintertime interactions between the CWP3 wavetrain and the North Atlantic storm track may, in their turn, influence post-winter Eurasian climate. Such a hypothesis is suggested by a possible linkage of the wintertime NAO to a wavenumber-5 teleconnection along the subsequent summertime polar front jet between the British Isles and Lake Baikal^61^. Verification of this hypothesis requires further investigations. Further in-depth investigations are also needed for a better understanding of mechanisms generating the CWP3 wavetrain itself and its role in bridging the wintertime surface climate variability in the Euro-Atlantic region and northern Asia. The next step may involve time-lagged covariance analyses of key variables from different reanalysis products or climate models. Experiments with an atmospheric general circulation model run with different boundary conditions and/or parameterisations should also be helpful.

## Methods

### Basic and derived data

Atmospheric variability in the ESO period is studied using monthly and daily mean fields on a longitude-latitude grid of 2.5° × 2.5° from the NCEP/NCAR reanalysis^5^ (obtained via http://www.esrl.noaa.gov/psd/). Only data from the winter (December-March) season are analysed. The monthly mean fields of the surface air temperature (SAT), sea level pressure (SLP) and surface wind velocity u_*s*_ are converted into winter (DJFM) means. The same procedure is applied to the pressure vertical velocity ${\rm{\omega }}$ (positive downward) at 12 constant pressure levels (from 1000 to 100 hPa). Winter means are also computed for the air temperature *T*, potential temperature *θ*, geopotential height (denoted as GPH or *Z*) and horizontal wind velocity **u**. The zonal (*u*) and meridional (*v*) components of **u** are positive in the eastward and northward directions, respectively. The fields of *T*, *θ*, *Z* and **u** are provided at 17 constant pressure levels (from 1000 to 10 hPa). The potential temperature is used to calculate the buoyancy frequency, a common measure of static stability^45^, which in the log-pressure coordinate *z* reads as

1 $$N={(\frac{g}{\theta }\frac{\partial \theta }{\partial z})}^{1/2},$$

where *g* and $\partial /\partial z$ stand for the gravitational acceleration and the vertical differentiation operator, respectively. The log-pressure coordinate is expressed in terms of the pressure *p* at a given level as $z=-\,H\,\mathrm{ln}(p/{p}_{s})$, where *p*_*s*_ is the pressure at the surface (assumed equal to 1000 hPa) and *H* is the standard scale height (assumed equal to 8500 m). The horizontal wind velocity is used to compute the vertical component of the relative vorticity defined as $\zeta ={\bf{k}}\cdot {\nabla }_{h}\times {\bf{u}}$, where **k** is a vertical unit vector, and ${\nabla }_{h}$ is the horizontal gradient operator. Following the related study^8^, a schematic of wintertime jet streams is obtained by finding, on each meridian, the latitudes of up to three largest local maxima of the climatological wind speed at the 500-hPa level and plotting the 300-hPa climatological wind vectors at these latitudes (arrows in Figs 2c, 6a and 8a).

To analyse anomalous wintertime heat budgets, the following linearised thermodynamic equation is used^62,63^:

2 $$0\approx \frac{\partial {\theta }_{a}}{\partial t}=-\,{{\bf{u}}}_{a}\cdot {\nabla }_{h}{\theta }_{m}-{{\bf{u}}}_{m}\cdot {\nabla }_{h}{\theta }_{a}-{\omega }_{a}\frac{\partial {\theta }_{m}}{\partial p}+{E}_{a}+{J}_{a},$$

where *t* is the time, subscripts *m* and *a* denote the climatological mean of the given wintertime variable and departure (anomaly) from this mean, respectively, *E*_*a*_ is the anomaly of the transient eddy heat flux convergence, and *J*_*a*_ is the anomalous diabatic heating. The sum *E*_*a*_ + *J*_*a*_ is estimated as the residual of the other terms.

To diagnose quasi-stationary Rossby waves, the horizontal component ${{\bf{F}}}_{s}^{(h)}$ and the vertical component ${F}_{s}^{(z)}$ of the Plumb wave activity flux **F**_*s*_^51^ is calculated as follows:

3 $${{\bf{F}}}_{s}^{(h)}={p}_{r}[{v}^{\ast }{v}^{\ast }-\tfrac{g}{2f\,R\,\cos \,\varphi }\tfrac{\partial ({v}^{\ast }{Z}^{\ast })}{\partial \lambda },-\,{u}^{\ast }{v}^{\ast }+\tfrac{g}{2f\,R\,\cos \,\varphi }\tfrac{\partial ({u}^{\ast }{Z}^{\ast })}{\partial \lambda }]\,\cos \,\varphi ,$$

4 $${F}_{s}^{(z)}={p}_{r}\frac{f}{S}[{v}^{\ast }{T}^{\ast }-\tfrac{g}{2f\,R\,\cos \,\varphi }\tfrac{\partial ({T}^{\ast }{Z}^{\ast })}{\partial \lambda }]\,\cos \,\varphi ,$$

where *p*_*r*_ denotes the ratio of *p* and *p*_*s*_, the asterisk in superscript indicates deviation from the zonal mean, *f* is the Coriolis parameter, *R* is the earth’s radius, *λ* and $\varphi$ are the longitude and latitude, respectively, and *S* is the static stability of the basic state. The latter is defined as $S=\partial {\hat{T}}_{m}/\partial z+\kappa {\hat{T}}_{m}/H$, where ${\hat{T}}_{m}$ is the climatological mean temperature averaged over the area north of 20°N and $\kappa$ is the Poisson constant. The divergence of the wave activity flux indicates local sources and sinks of stationary wave activity. Its zonal mean scaled by the factor ${({p}_{r}\cos \varphi )}^{-1}$ can be written as

5 $${D}_{F}=\mathop{\underbrace{\frac{1}{R\,{\cos }^{2}\,\varphi }\frac{\partial }{\partial \varphi }[{\cos }^{2}\,\varphi \{\,-\,{u}^{\ast }{v}^{\ast }\}]}}\limits_{{D}_{F}^{(y)}}+\mathop{\underbrace{\frac{1}{{p}_{r}}\frac{\partial }{\partial z}[{p}_{r}\frac{f}{S}\{{v}^{\ast }{T}^{\ast }\}]}}\limits_{{D}_{F}^{(z)}},$$

where the braces denote zonal averaging. ${D}_{F}^{(y)}$ and ${D}_{F}^{(z)}$ are the contributions to *D*_*F*_ from the meridional and vertical divergence of the zonally-averaged **F**_*s*_ vector, respectively. Here, the components of **F**_*s*_ are calculated using the winter mean data of *u*, *v*, *Z*, and *T*.

Storm track variations are investigated using the seasonal variance of the high-pass filtered daily mean meridional wind at 300 hPa. This variance is symbolised ${\overline{v^{\prime} v^{\prime} }}_{300}$, where the overbar denotes the average over the winter season, the prime indicates filtering, and the subscript refers to the pressure level. As in the related study^8^, a filter with seven weights ([−1, −3, −5, 18, −5, −3, −1]/24) is applied. It retains synoptic variability between two and six days^46^. For comparison’s sake, monthly variances of ${\overline{v^{\prime} v^{\prime} }}_{300}$ in the winter (D,J,F,M) months (a total of 4 × 38 months in the ESO period) are also calculated. These variances and monthly means of some other variables are used to construct month-to-month anomalies (obtained by subtracting the annual cycle from the monthly mean data).

The DJFM variances and covariances of $v^{\prime}$, $u^{\prime}$ and $T^{\prime}$ are also used to express the storm track activity in terms of the **E**-vectors devised by Trenberth^46^. These vectors can be written as

6 $${{\bf{E}}}_{u}=\mathop{\underbrace{[\frac{1}{2}(\overline{{v^{\prime} }^{2}}-\overline{{u^{\prime} }^{2}}),-\,\overline{u^{\prime} v^{\prime} }}}\limits_{{{\bf{E}}}_{u}^{(h)}},\mathop{\underbrace{f\frac{\overline{v^{\prime} T^{\prime} }}{S}]}}\limits_{{E}_{u}^{(z)}}\,\cos \,\varphi ,$$

and

7 $${{\bf{E}}}_{v}=\mathop{\underbrace{[-\overline{u^{\prime} v^{\prime} },-\,\frac{1}{2}(\overline{{v^{\prime} }^{2}}-\overline{{u^{\prime} }^{2}})}}\limits_{{{\bf{E}}}_{v}^{(h)}},\mathop{\underbrace{-f\frac{\overline{u^{\prime} T^{\prime} }}{S}]}}\limits_{{E}_{v}^{(z)}}\,\cos \,\varphi .$$

In Eq. 6, ${{\bf{E}}}_{u}^{(h)}$ and ${E}_{u}^{(z)}$ mark the horizontal and vertical components of the **E**_*u*_ vector. In Eq. 7, ${{\bf{E}}}_{v}^{(h)}$ and ${E}_{v}^{(z)}$ mark the corresponding components of the **E**_*v*_ vector. The vector ${{\bf{E}}}^{(z)}=({E}_{u}^{(z)},{E}_{v}^{(z)})$ provides a compact representation of baroclinic synoptic processes. The **E**_*u*_ vector approximately points in the direction of the synoptic wave energy propagation. The divergences of ${p}_{r}{{\bf{E}}}_{u}$ and ${p}_{r}{{\bf{E}}}_{v}$ scaled by the factor ${({p}_{r}\cos \varphi )}^{-1}$ represent local forcing in, respectively, the zonal and meridional components of the transformed momentum equation for a quasi-geostrophic motion (see ref.^46^ for details). When this equation is zonally-averaged, the forcing of the zonal-mean zonal flow by the synoptic eddies reads as

8 $${D}_{E}=\mathop{\underbrace{\frac{1}{R\,{\cos }^{2}\,\varphi }\frac{\partial }{\partial \varphi }[{\cos }^{2}\,\varphi (\,-\,\overline{u^{\prime} v^{\prime} })]}}\limits_{{D}_{E}^{(y)}}+\mathop{\underbrace{\frac{1}{{p}_{r}}\frac{\partial }{\partial z}[{p}_{r}\frac{f}{S}\overline{v^{\prime} T^{\prime} }]}}\limits_{{D}_{E}^{(z)}},$$

where ${D}_{E}^{(y)}$ and ${D}_{E}^{(z)}$ are the contributions to *D*_*E*_ from the meridional and vertical divergence of the zonally-averaged **E**_*u*_ vector, respectively.

### Statistical analysis

Dominant modes of interannual variability in a given region are obtained via the Empirical Orthogonal Function (EOF) decomposition of field anomalies^64^. The anomalies are computed by subtracting the local linear trend from the wintertime data at each grid point. The convergence of meridians is taken into account by weighting the anomalies by the factor ${\cos }^{1/2}\,\varphi$. The principal component (PC) time series from the EOF decomposition are standardised to have zero mean and a standard deviation of one. The anomalies of selected fields are then regressed onto the PC of the first leading mode of a given variable.

The first modes of extratropical variability in sea level pressure are computed separately for a North Atlantic domain (Fig. 1a, magenta box), extending from 20°N to 80°N and from 90°W to 40°E, and an Eurasian domain (Fig. 2b, magenta box) extending from 30°N to 80°N and from 10°E to 140°E. These modes are referred to as the NAO mode and the SLP_EA_ mode, respectively. The NAO mode is a version of the standard EOF-based NAO mode^4^, calculated over the same domain as in the related study^8^. The first mode of extratropical variability in storm track activity (${\overline{v^{\prime} v^{\prime} }}_{300}$) is computed for the Eurasian domain. It is the STA_EA_ mode introduced in the related study^8^. The NAO, SLP_EA_ and STA_EA_ modes explain 52.4%, 58.6% and 28.2% of the variance in the data, respectively. All these modes are statistically reliable according to North’s “rule of thumb” employed to assess their uniqueness from uncertainty on the eigenvalues of the covariance matrix^65^.

The PC time series of the NAO, SLP_EA_ and STA_EA_ modes are referred to as the NAO, SLP_EA_ and STA_EA_ indices, respectively. Additional indices of interannual variability are constructed by area-averaging of selected variables. The GPH_LB_ index, introduced in the related study^8^, is obtained by averaging the geopotential height at 300 hPa in the Lake Baikal area (Fig. 2c, blue box) extending from 45°N to 60°N and from 90°E to 125°E. The SAT_E_ and SAT_A_ indices are obtained by averaging the surface air temperature over northern Europe (Fig. 1b, blue box) and northern Asia (Fig. 1b, magenta box). The European domain extends from 40°N to 70°N and from 0°E to 60°E. The Asian domain extends from 40°N to 70°N and from 60°E to 140°E (approximately the same area as used for computation of the corresponding SAT_A_ index in the related study^8^). The SAT_E+A_ index is obtained by averaging the surface air temperature over the combined European and Eurasian domains. The linearly detrended and standardised time series of indices used in regressions are shown in Supplementary Fig. S1.

Relations between time series are quantified using the sample correlation coefficient *r*. A two-sided Student’s *t*-test^64^ is employed to assess statistical significance of *r*. The test is performed using an effective sample size^66^ defined as ${N}_{eff}={N}_{0}(1-{r}_{a}{r}_{b})/(1+{r}_{a}{r}_{b})$, where *N*_0_ is the length of the series while ${r}_{a}$ and ${r}_{b}$ are the lag-one autocorrelations of the correlated series *a* and *b*. Correlation coefficients between the “thermodynamic” indices (SAT_E_, SAT_A_ and SAT_E+A_) and “dynamic” indices (NAO, SLP_EA_, GPH_LB_ and STA_EA_) are given in Table 1. In regression plots (Figs 1–11), the anomalies of scalar fields significant at the 95% confidence level are shaded and the anomalies of vector fields are plotted at points at which the anomaly of any vector component is significant at the 95% confidence level.

## Supplementary information

Supplementary Figures and Table
