# Supplementary material for: Upper-tropospheric bridging of wintertime surface climate variability in the Euro-Atlantic region and northern Asia
Source: Sci Rep. 2019 Oct 10;9:14660. doi: 10.1038/s41598-019-51019-w (PMC6787261; doi:10.1038/s41598-019-51019-w)
Supplement: Supplementary file 1 — Supplementary Figures and Table [file 41598_2019_51019_MOESM1_ESM.pdf]

# Upper-tropospheric bridging of wintertime surface climate variability in the Euro-Atlantic region and northern Asia

Pawel Schlichtholz
Institute of Oceanology
Polish Academy of Sciences
Powstancow Warszawy 55
81-712 Sopot, Poland
e-mail: schlicht@iopan.gda.pl

September 30, 2019

This document contains the following supplementary information:

**(i)** Supplementary Figures S1-S3 (pages 2-4)

**(ii)** Supplementary Table S1 (page 5)

# (i) Supplementary Figures

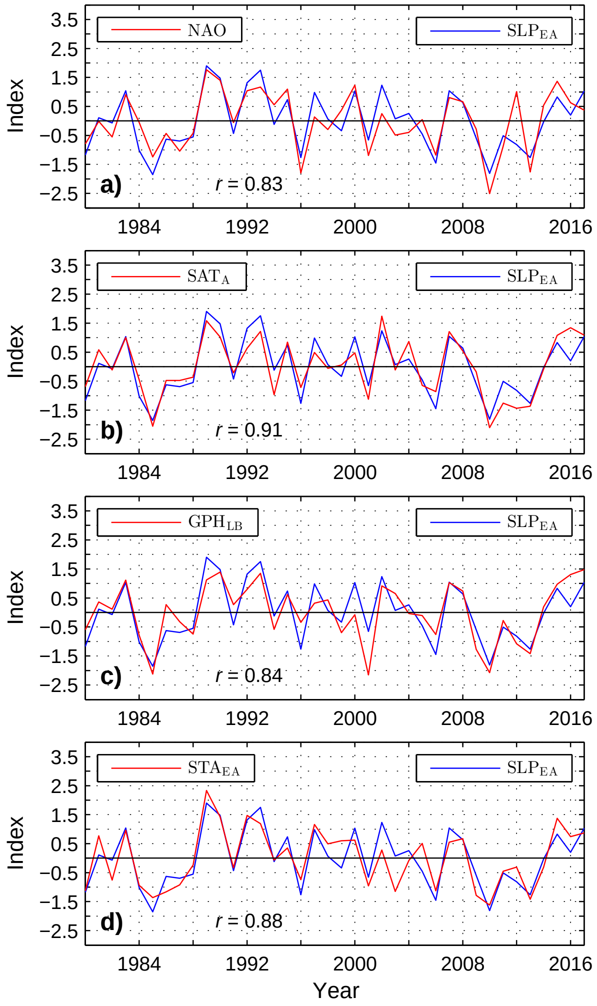


Figure S1: **Comparison of the $\mathrm{SLP_{EA}}$ index (blue curves) with other indices (red curves) of the wintertime (DJFM mean) climate variability in the Eurasian region during the ESO period.** The $\mathrm{SLP_{EA}}$ index is defined as the first principal component time series of the sea level pressure (SLP) variability over extratropical Eurasia (30°-80°N, 10°-140°E). The other indices are (**a**) the NAO index defined as the first principal component time series of the SLP variability in the North Atlantic region (20°-80°N, 90°W-40°E), (**b**) the $\mathrm{SAT_A}$ index defined as the time series of the surface air temperature averaged over northern Asia (40°-70°N, 60°-140°E), (**c**) the $\mathrm{GPH_{LB}}$ index defined as the time series of the geopotential height at 300 hPa averaged over the Lake Baikal area (45°-60°N, 90°-125°E) and (**d**) the $\mathrm{STA_{EA}}$ index defined as the first principal component time series of the storm track activity ($\overline{v'v'}_{300}$) variations over extratropical Eurasia (30°-80°N, 10°-140°E). All indices are based on linearly detrended data and standardised to have a mean of 0 and a standard deviation of 1. Years correspond to the January. In (**a-d**) the indicated value of $r$ is the correlation coefficient between the given index and the $\mathrm{SLP_{EA}}$ index. All correlations are significant at the 99.99% confidence level.

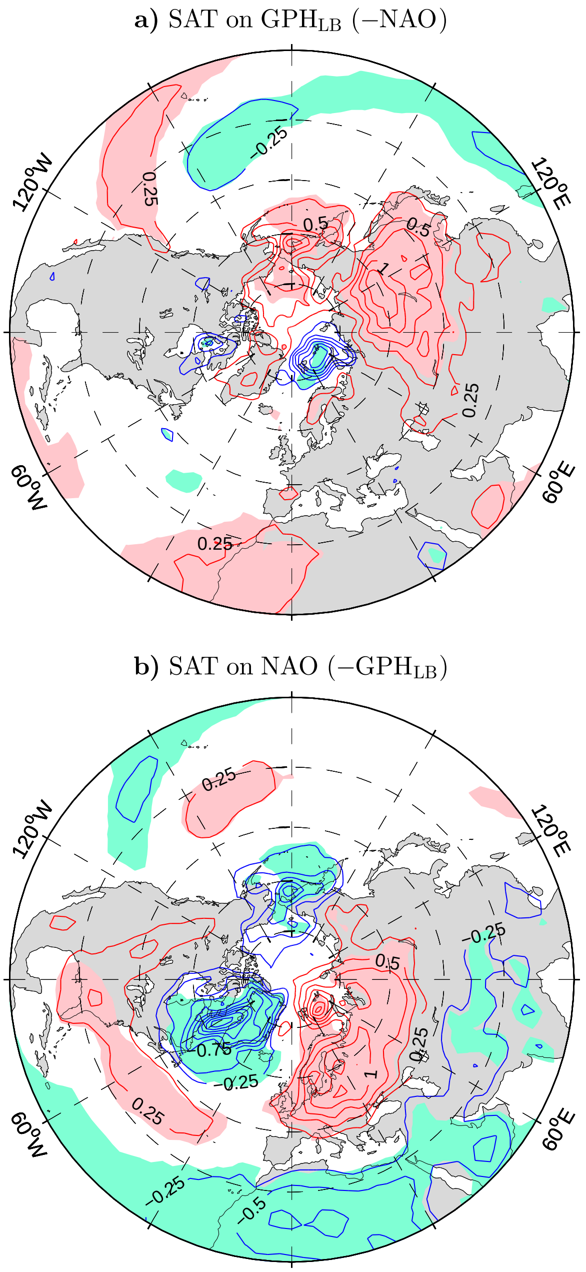


Figure S2: **Relation of wintertime (DJFM mean) anomalies of surface air temperature (SAT) to the GPH$_{LB}$ and NAO indices in the ESO period.** (**a**) Detrended anomalies of SAT regressed onto the GPH$_{LB}$ index (red curve in Supplementary Fig. 1c) after removing the signal associated with the NAO index (red curve in Supplementary Fig. 1a). The CI is 0.25 K per 1 standard deviation of the NAO-independent contribution to the GPH$_{LB}$ index. (**b**) Detrended anomalies of SAT regressed onto the NAO index after removing the signal associated with the GPH$_{LB}$ index. The CI is 0.25 K per 1 standard deviation of the GPH$_{LB}$-independent contribution to the NAO index. In (**a** and **b**) red and blue contours represent positive and negative anomalies, respectively. Pink and aquamarine shading denote, respectively, positive and negative anomalies statistically significant at the 95% confidence level. The maps were generated by MathWorks MATLAB R2014a with M_Map (http://www.eoas.ubc.ca/~rich/map.html).

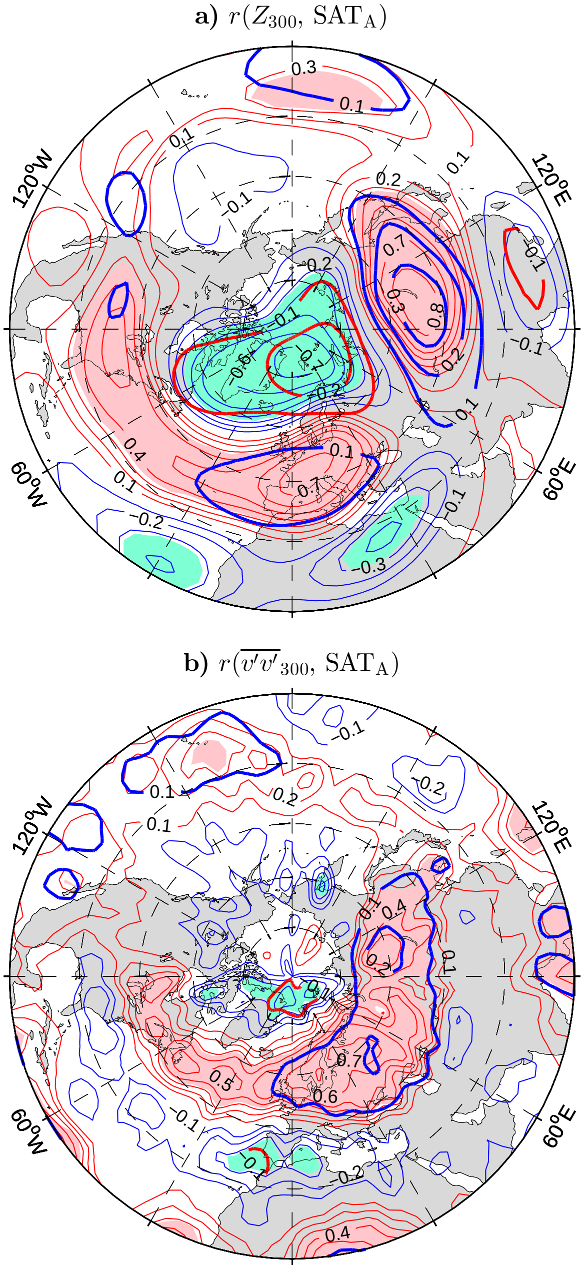


Figure S3: **Relation of upper-tropospheric anomalies to indices of surface air temperature variability in northern Asia during the ESO period.** (**a**) Correlation coefficients for the winter (DJFM) mean anomalies (thin contours) and month-to-month anomalies in the winter (D,J,F,M) months (thick contours) of the geopotential height at 300 hPa with the corresponding $\mathrm{SAT_A}$ indices defined as anomalies of surface air temperature averaged over northern Asia (40°-70°N, 60°-140°E). The CI is 0.1. Pink (aquamarine) shading denotes positive (negative) correlations statistically significant at the 95% confidence level for the winter mean data. (**b**) As (**a**) but for the correlation coefficients of the $\mathrm{SAT_A}$ indices with the corresponding anomalies of the upper-tropospheric storm track activity ($\overline{v'v'}_{300}$). In (**a** and **b**) the month-to-month anomalies are defined as monthly averaged departures from the annual cycle. The maps were generated by MathWorks MATLAB R2014a with M_Map (http://www.eoas.ubc.ca/~rich/map.html).

# (ii) Supplementary Table

Table S1: **Main acronyms, abbreviations, and indices.**

| Acronym | Explanation |
|---|---|
| AO | Arctic Oscillation |
| CWP3 | wavenumber-3 circumglobal waveguide pattern |
| DJFM | winter (December-to-March) |
| EOF | empirical orthogonal function |
| ESO | era of satellite observations; here the 1979-2017 period |
| GPH | geopotential height |
| $\mathrm{GPH_{LB}}$ | GPH at 300 hPa ($Z_{300}$) averaged over the Lake Baikal area (LB box in Fig. 2c) |
| NAO | North Atlantic Oscillation |
| NAO index | PC1 of SLP variability in the North Atlantic region (NA box in Fig. 1a) |
| NCEP/NCAR | National Centers for Environmental Prediction/National Center for Atmospheric Research |
| NH | Northern Hemisphere |
| PC | principal component |
| PC1 | PC of the first EOF mode |
| SAT | surface air temperature |
| $\mathrm{SAT_{A}}$ | SAT averaged over northern Asia (A box in Fig. 1b) |
| $\mathrm{SAT_{E}}$ | SAT averaged over Europe (E box in Fig. 1b) |
| $\mathrm{SAT_{E+A}}$ | SAT averaged over northern Eurasia (E and A boxes in Fig. 1b) |
| SLP | sea level pressure |
| $\mathrm{SLP_{EA}}$ index | PC1 of SLP variability over extratropical Eurasia (EA box in Fig. 2b) |
| STA | storm track activity ($\overline{v'v'}_{300}$) |
| $\mathrm{STA_{EA}}$ index | PC1 of STA variability over extratropical Eurasia (EA box in Fig. 6b) |
